# Supplementary material for: Identification and 3D modeling of bioactive peptides from Lactobacillus brevis RAMULAB49 protein hydrolysate with in silico ERK1 phosphorylation inhibition activity targeting diabetic nephropathy
Source: PLoS One. 2025 Sep 22;20(9):e0331192. doi: 10.1371/journal.pone.0331192 (PMC12453238; doi:10.1371/journal.pone.0331192)
Supplement: S1 File — (DOCX) [file pone.0331192.s001.docx]

**S1.1. Table:** **LC-MS/MS results of peptides identified from *Lactobacillus brevis* RAMULAB49 protein hydrolysates.**

| **Pepsin** | | | | | | | | | | | | | | | | |
| --- | --- | --- | --- | --- | --- | --- | --- | --- | --- | --- | --- | --- | --- | --- | --- | --- |
| **Master Protein** | **Accession** | **Coverage [%]** | **Peptides** | **Sequence** | **MW [Da]** | **Position** | **No of AA** | **GRAVY** | **pI** | **XCorr** | **m/z [Da]** | **MH+ [Da]** | **Theo.MH+ [Da]** | **DeltaM [PPM]** | **Deltam/z [Da]** | **RT [min]** |
| 2,4-diacetylphloroglucinol biosynthesis protein OS=Limosilactobacillus reuteri OX=1598 GN=rtcPhlb PE=4 SV=1 | A0A073JRP2 | 10 | 1 | [K].QSRSSNTSWKYGYK.[F] | 1691.82 | 126-139 | 14 | -1.936 | 10.33 | 2.81 | 564.612 | 1691.821 | 1691.814 | 0.0356 | 0.00261 | 15.201 |
| 2-deoxyuridine 5-triphosphate nucleotidohydrolase OS=Lactobacillus sp. OX=1591 GN=DUD35_03755 PE=4 SV=1 | A0A425XPG0 | 11 | 1 | [K].FGLVDFGYTEQEIQDAFNDK.[N] | 2336.5 | 148-167 | 20 | -0.6 | 3.77 | 1.98 | 1168.543 | 2336.079 | 2336.072 | 0.1162 | 0.00359 | 26.1378 |
| 33 kDa chaperonin OS=Lactiplantibacillus pentosus OX=1589 GN=hslO PE=3 SV=1 | A0A2K9I441 | 5 | 1 | [R].AYAVDATGVVAEAQQR.[H] | 1648.79 | 17-32 | 16 | 0.044 | 4.37 | 2.39 | 550.2847 | 1648.84 | 1648.829 | 0.0586 | 0.00357 | 17.1848 |
| Acetoin ABC transporter ATP-binding protein OS=Dellaglioa algida OX=105612 GN=LABALGLTS371_14290 PE=4 SV=1 | A0A5C6M6W8 | 7 | 1 | [K].EKLFYLDTQNNFFDSYTIDK.[I] | 2501.73 | 74-93 | 20 | -0.83 | 4.23 | 2.51 | 834.3939 | 2501.167 | 2501.187 | 0.1155 | -0.00669 | 17.7323 |
| Acetyl-CoA carboxytransferase OS=Secundilactobacillus mixtipabuli OX=1435342 GN=accA PE=4 SV=1 | A0A1Z5IDJ8 | 4 | 1 | [R].LSNRAAFFR.[R] | 1081.24 | 242-250 | 9 | -0.033 | 12 | 2.31 | 361.2001 | 1081.586 | 1081.59 | 0.2251 | -0.00143 | 14.9951 |
| Alcohol dehydrogenase OS=Companilactobacillus alimentarius DSM 20249 OX=1423720 GN=LA20249_07245 PE=4 SV=1 | A0A2K9HNV5 | 4 | 1 | [K].LGADATVPFDMTTK.[D] | 1466.67 | 173-186 | 14 | 0.093 | 4.21 | 2.08 | 741.8618 | 1482.716 | 1482.714 | 1.19 | 0.00088 | 17.2087 |
| Alpha/beta hydrolase OS=Lactobacillus crispatus OX=47770 GN=F8251_10135 PE=4 SV=1 | A0A6A1Z0E4 | 7 | 1 | [K].SLKDYTVADEAAELAK.[I] | 1723.9 | 65-80 | 16 | -0.35 | 4.32 | 2.47 | 575.2941 | 1723.868 | 1723.875 | -4.08 | -0.00235 | 21.9015 |
| Aminotransferase OS=Bombilactobacillus bombi OX=1303590 GN=DS831_01270 PE=3 SV=1 | A0A3R6WBE7 | 2 | 1 | [RK].LADYLNK.[-V] | 835.96 | 381-387 | 7 | -0.4 | 5.83 | 1.99 | 418.7295 | 836.4518 | 836.4512 | 0.63 | 0.00026 | 17.7135 |
| Cell division protein FtsZ OS=Ligilactobacillus salitolerans OX=1808352 GN=ftsZ PE=3 SV=1 | A0A401IR21 | 7 | 1 | [K].TVMQDQGSALMGIGTANGENRTEEATK.[K] | 2810.06 | 218-244 | 27 | -0.737 | 4.41 | 2.09 | 1405.157 | 2809.307 | 2809.309 | -0.77 | -0.00108 | 27.0409 |
| Citrate (pro-3S)-lyase subunit beta OS=Limosilactobacillus fermentum OX=1613 GN=citE PE=3 SV=1 | A0A1Q2T492 | 12 | 1 | [R].TMMFVPGNNAGMVKDAGIYGADSIMFDLEDSVSMSEK.[D] | 3959.5 | 8-44 | 37 | 0.046 | 3.96 | 3.33 | 1335.912 | 4005.722 | 4005.757 | -8.82 | -0.01177 | 29.205 |
| DEAD/DEAH box helicase family protein OS=Lactobacillus panisapium OX=2012495 GN=GYM71_05080 PE=4 SV=1 | A0A8G0AED3 | 1 | 1 | [K].VTDLDLTAEVVK.[S] | 1302.49 | 335-346 | 10 | 0.517 | 4.03 | 2.25 | 651.8627 | 1302.718 | 1302.715 | 2.26 | 0.00147 | 28.8403 |
| DEAD-box ATP-dependent RNA helicase CshA OS=Bombilactobacillus bombi OX=1303590 GN=cshA PE=3 SV=1 | A0A3R6Z9U4 | 4 | 1 | [R].GGNNRNSNHGASRDNSHGR.[A] | 2006.99 | 467-485 | 19 | -2.268 | 11.7 | 2.31 | 669.6434 | 2006.916 | 2006.9 | 7.94 | 0.00531 | 15.8001 |
| DEAD-box ATP-dependent RNA helicase CshA OS=Carnobacterium divergens OX=2748 GN=cshA PE=3 SV=1 | A0A5F0ML74 | 2 | 1 | [K].SGGNRGKATDNR.[R] | 1232.28 | 518-529 | 12 | -2.025 | 10.83 | 1.96 | 616.8023 | 1232.597 | 1232.609 | -9.49 | -0.00585 | 16.2252 |
| Deaminase OS=Companilactobacillus suantsaicola OX=2487723 GN=EGT49_10940 PE=4 SV=1 | A0A4Z0JFI5 | 9 | 1 | [-].MKKPYIICHMMTSVDGR.[I] | 2010.48 | 1-17 | 17 | -0.141 | 9.19 | 2.02 | 1042.009 | 2083.01 | 2082.996 | 6.96 | 0.00725 | 26.5479 |
| DHHA1 domain protein OS=Limosilactobacillus reuteri (strain ATCC 55730 / SD2112) OX=491077 GN=HMPREF0538_21760 PE=4 SV=1 | F8DN32 | 10 | 1 | [K].IDHHPNDEPFGDIMWVEPDASSCSEMIYSFYQR.[F] | 3917.27 | 127-159 | 33 | -0.694 | 4.14 | 3.03 | 1330.236 | 3988.693 | 3988.689 | 1.15 | 0.00153 | 29.2075 |
| DNA mismatch repair protein MutS OS=Furfurilactobacillus siliginis OX=348151 GN=mutS PE=3 SV=1 | A0A0R2L0X9 | 2 | 1 | [K].QMNLMAMTPMDVMNK.[F] | 1755.17 | 847-861 | 15 | -0.06 | 5.84 | 2.15 | 885.8947 | 1770.782 | 1770.772 | 5.99 | 0.0053 | 26.0443 |
| DNA polymerase III PolC-type OS=Companilactobacillus alimentarius DSM 20249 OX=1423720 GN=polC PE=3 SV=1 | A0A2K9HPP1 | 1 | 1 | [K].QAMKDANVPDWYIDSCLKIK.[Y] | 2338.72 | 1237-1256 | 20 | -0.44 | 6.03 | 2.85 | 1206.092 | 2411.176 | 2411.173 | 1.02 | 0.00123 | 26.3911 |
| DNA/RNA non-specific endonuclease OS=Lacticaseibacillus rhamnosus OX=47715 GN=HWN39_14440 PE=4 SV=1 | A0A7Y7UK16 | 6 | 1 | [K].TNSAISSLDGAIADKR.[E] | 1618.76 | 48-63 | 16 | -0.263 | 5.63 | 2.02 | 809.9197 | 1618.832 | 1618.839 | -4.58 | -0.0037 | 30.4358 |
| DUF5776 domain-containing protein OS=Apilactobacillus kunkeei OX=148814 GN=RZ73_02060 PE=4 SV=1 | A0A0M9D6S9 | 0 | 2 | [K].AGTSFTIGSFNGDGWNSIK.[N] | 1959.1 | 1163-1181 | 19 | -0.226 | 5.88 | 2.47 | 653.6401 | 1958.906 | 1958.924 | -9.41 | -0.00614 | 15.2641 |
| Flavodoxin OS=Ligilactobacillus saerimneri OX=228229 GN=GTO87_00150 PE=4 SV=1 | A0A7H9EIY4 | 12 | 1 | [K].DHAQVVPGYRYDGDKQGMR.[K] | 2192.39 | 134-152 | 19 | -1.416 | 6.75 | 2.44 | 731.346 | 2192.023 | 2192.03 | -3.13 | -0.00229 | 16.0071 |
| Glutaminase OS=Lactobacillus hominis DSM 23910 = CRBIP 24.179 OX=1423758 GN=glsA PE=3 SV=1 | I7JUU9 | 6 | 1 | [K].SLMMTTGLYNESGVYSAR.[I] | 1980.24 | 233-250 | 19 | -0.072 | 5.72 | 2.67 | 990.4656 | 1979.924 | 1979.92 | 1.9 | 0.00188 | 27.3784 |
| Glycoside hydrolase family 65 protein OS=Lactobacillus sp. OX=1591 GN=DUD28_04990 PE=3 SV=1 | A0A425X6P8 | 2 | 1 | [K].YRGRQIDIDINQK.[T] | 1618.81 | 720-732 | 13 | -1.431 | 8.59 | 2.43 | 809.9381 | 1618.869 | 1618.866 | 1.76 | 0.00142 | 27.2232 |
| GNAT family N-acetyltransferase OS=Ligilactobacillus salivarius OX=1624 GN=B6U56_07075 PE=4 SV=1 | A0A1V9R3B5 | 11 | 1 | [K].YYRKFGFVSGQSMNIYYDPFPR.[N] | 2736.1 | 129-150 | 22 | -0.691 | 9.4 | 2.56 | 912.4316 | 2735.28 | 2735.308 | -9.92 | -0.00904 | 26.1353 |
| GRAM_POS_ANCHORING domain-containing protein OS=Limosilactobacillus reuteri OX=1598 GN=HF82_03830 PE=4 SV=1 | A0A073JNZ7 | 2 | 1 | [K].VTQGSINFAKSVAENYK.[N] | 1856.07 | 375-391 | 17 | -0.382 | 8.47 | 2.58 | 619.3189 | 1855.942 | 1855.955 | -6.83 | -0.00423 | 26.8524 |
| GTPase Der OS=Finegoldia magna ACS-171-V-Col3 OX=768713 GN=der PE=3 SV=1 | D9PRF3 | 4 | 1 | [R].MIVTDIAGTTRDAIDSK.[I] | 1807.05 | 202-218 | 17 | 0.059 | 4.43 | 2.05 | 911.9647 | 1822.922 | 1822.922 | 0.36 | 0.00033 | 30.6012 |
| HTH lysR-type domain-containing protein OS=Limosilactobacillus fermentum OX=1613 GN=LACFE_CDS1035 PE=3 SV=1 | A0A1D7ZXF1 | 5 | 1 | [R].QAPQLVQDFMNR.[L] | 1446.64 | 242-253 | 12 | -0.758 | 5.84 | 1.96 | 723.8573 | 1446.707 | 1446.716 | -5.87 | -0.00424 | 16.0729 |
| Lactobacillus shifted protein OS=Golovinomyces cichoracearum OX=62708 GN=GcM1_238096 PE=4 SV=1 | A0A420IJG7 | 6 | 1 | [K].SQNPRSRAMSGPR.[F] | 1443.6 | 89-101 | 13 | -1.754 | 12.3 | 2.4 | 487.2401 | 1459.706 | 1459.718 | -8.55 | -0.00416 | 15.8174 |
| PDZ domain-containing protein OS=Lactobacillus sp. OX=1591 GN=DUD28_00010 PE=3 SV=1 | A0A3R8NHA7 | 3 | 1 | [K].YPSYAYLPMVSDQK.[T] | 1661.89 | 389-402 | 14 | -0.564 | 5.83 | 2.09 | 831.3923 | 1661.777 | 1661.788 | -6.37 | -0.00529 | 25.853 |
| Peptidase_M16_C domain-containing protein OS=Ligilactobacillus agilis OX=1601 GN=BEN83_06890 PE=4 SV=1 | A0A222W4Y6 | 5 | 1 | [R].QDSQATALIQATMDQLSQSK.[V] | 2036.24 | 363-382 | 19 | -0.489 | 4.21 | 2.37 | 1082.532 | 2164.056 | 2164.055 | 0.42 | 0.00046 | 30.0476 |
| Peptide ABC transporter substrate-binding protein OS=Ligilactobacillus agilis OX=1601 GN=BEN83_02840 PE=4 SV=1 | A0A222W2V4 | 3 | 1 | [K].INEFIQSQLEDNLGSK.[K] | 1835 | 396-411 | 16 | -0.688 | 4.14 | 2.17 | 917.9578 | 1834.908 | 1834.918 | -5.39 | -0.00494 | 30.8595 |
| Peptide ABC transporter substrate-binding protein OS=Liquorilactobacillus hordei OX=468911 GN=BSQ49_06940 PE=4 SV=1 | A0A3S6QPH9 | 5 | 1 | [K].VLGDGSTAASSFVSPETATDPTTGK.[D] | 2396.55 | 327-351 | 25 | -0.204 | 4.03 | 2.93 | 799.3884 | 2396.151 | 2396.146 | 1.75 | 0.0014 | 18.7828 |
| Phage terminase OS=Limosilactobacillus fermentum OX=1613 GN=LFER_1163 PE=4 SV=1 | A0A158SMI5 | 2 | 1 | [K].FASIAPNVDTGEPTK.[L] | 1546.7 | 84-98 | 15 | -0.34 | 4.37 | 2.31 | 516.2601 | 1546.766 | 1546.775 | -5.89 | -0.00303 | 18.947 |
| Pseudouridine synthase OS=Schleiferilactobacillus shenzhenensis LY-73 OX=1231336 GN=yjbO PE=3 SV=1 | U4TWE4 | 2 | 1 | [R].LLAPPER.[G] | 794.95 | 60-66 | 7 | -0.257 | 6 | 2.12 | 398.2395 | 795.4717 | 795.4723 | -0.74 | -0.00029 | 16.9125 |
| PTS EIIA type-2 domain-containing protein OS=Lacticaseibacillus manihotivorans OX=88233 GN=LM010_01820 PE=4 SV=1 | A0A5P8JMF6 | 10 | 1 | [R].DDPVGLGMFADDLI.[-] | 1477.65 | 134-147 | 14 | 0.457 | 3.32 | 2.07 | 739.3433 | 1477.679 | 1477.688 | -5.79 | -0.00428 | 26.5065 |
| PTS Gat IIA OS=Lactobacillus melliventris OX=1218507 GN=JF74_03340 PE=4 SV=1 | A0A0F4LJS8 | 8 | 1 | [K].MLQKVADIVQNEK.[L] | 1515.79 | 118-130 | 13 | -0.377 | 5.82 | 2.24 | 766.4038 | 1531.8 | 1531.815 | -9.47 | -0.00726 | 30.5271 |
| PTS transporter subunit EIIA OS=Ligilactobacillus saerimneri OX=228229 GN=GTO87_08665 PE=4 SV=1 | A0A7H9EMU3 | 10 | 1 | [R].KSSLVTGQQLTGANK.[Q] | 1531.73 | 3-17 | 15 | -0.567 | 10 | 1.99 | 766.4216 | 1531.836 | 1531.844 | -5.13 | -0.00393 | 27.1144 |
| Putative glycosyltransferase, exosortase G system-associated OS=Lactiplantibacillus paraplantarum OX=60520 GN=ica2 PE=4 SV=1 | A0A512FHX6 | 4 | 1 | [R].TIESFCDQLFTMSGAFSAFR.[R] | 2258.55 | 209-228 | 20 | 0.415 | 4.37 | 2.4 | 777.6782 | 2331.02 | 2331.042 | -9.4 | -0.00731 | 15.81 |
| Putative membrane protein OS=Apilactobacillus kunkeei EFB6 OX=1419324 GN=LAKU_4c00030 PE=4 SV=1 | A0A836YVU5 | 0 | 1 | [K].AQKDAYNTGEANAEAGFDKTK.[Q] | 2229.34 | 3869-3889 | 21 | -1.329 | 4.78 | 2.66 | 743.6809 | 2229.028 | 2229.042 | -6.21 | -0.00461 | 16.6942 |
| Riboflavin biosynthesis protein OS=Secundilactobacillus folii OX=2678357 GN=ribF PE=3 SV=1 | A0A7X2XTH4 | 4 | 1 | [R].YKTTGTVIHGEAR.[G] | 1432.6 | 191-203 | 13 | -0.677 | 8.6 | 2.23 | 716.8791 | 1432.751 | 1432.754 | -2.37 | -0.0017 | 26.7731 |
| Ribonuclease R OS=Lactobacillus johnsonii OX=33959 GN=rnr PE=3 SV=1 | A0A1Z1NCX8 | 2 | 1 | [R].SVNDLKMTEFMADK.[V] | 1628.88 | 627-640 | 14 | -0.493 | 4.56 | 2.01 | 822.8893 | 1644.771 | 1644.761 | 6.39 | 0.00526 | 17.2637 |
| RNA polymerase sigma-54 factor OS=Lactobacillus pasteurii DSM 23907 = CRBIP 24.76 OX=1423790 GN=BN53_01415 PE=3 SV=1 | I7KKS9 | 3 | 1 | [K].LSPLSDDELVMEFR.[K] | 1650.86 | 378-391 | 14 | -0.1 | 3.92 | 2.48 | 556.2753 | 1666.811 | 1666.799 | 7.19 | 0.004 | 15.8848 |
| Single-stranded-DNA-specific exonuclease RecJ OS=Secundilactobacillus mixtipabuli OX=1435342 GN=recJ PE=3 SV=1 | A0A1Z5ICQ8 | 3 | 1 | [R].LDDAAPAVELLTTLDEERAEK.[L] | 2299.52 | 291-311 | 21 | -0.443 | 3.95 | 3.37 | 767.0635 | 2299.176 | 2299.166 | 4.22 | 0.00323 | 24.8416 |
| Site-specific integrase OS=Lactobacillus gasseri OX=1596 GN=BXT97_09480 PE=4 SV=1 | A0A1V3Y026 | 6 | 1 | [R].LGHSNMSTTASHYAYMLDELK.[Q] | 2369.65 | 335-355 | 21 | -0.419 | 5.99 | 2.85 | 790.3626 | 2369.073 | 2369.09 | -7.15 | -0.00565 | 16.1335 |
| T surface-antigen of pili OS=Secundilactobacillus pentosiphilus OX=1714682 GN=IWT25_01458 PE=4 SV=1 | A0A1Z5IWJ3 | 1 | 1 | [R].QDIDEETRDGGMKDGK.[L] | 1793.88 | 897-912 | 16 | -2.019 | 4.23 | 1.97 | 897.3958 | 1793.784 | 1793.797 | -7.07 | -0.00634 | 16.2489 |
| Tape measure protein OS=Ligilactobacillus saerimneri OX=228229 GN=GTO87_05725 PE=4 SV=1 | A0A7H9EKA3 | 0 | 1 | [K].LDALIEK.[DFRK] | 800.95 | 1459-1465 | 7 | 0.429 | 4.37 | 1.95 | 401.2391 | 801.4709 | 801.4717 | -0.96 | -0.00039 | 20.8795 |
| TatD family hydrolase OS=Ligilactobacillus ruminis OX=1623 GN=LRP_1085 PE=4 SV=1 | A0A0G8G5V3 | 9 | 1 | [K].TDAAKNGGIMHSFNGNAAWAEK.[F] | 2290.49 | 143-164 | 22 | -0.632 | 6.42 | 2.82 | 573.2665 | 2290.044 | 2290.067 | -9.98 | -0.00571 | 15.0051 |
| Tetratricopeptide repeat protein OS=Lactobacillus sp. ESL0233 OX=2069354 GN=F5ESL0233_08110 PE=4 SV=1 | A0A3M0MPX7 | 3 | 1 | [K].TISEVNSLMGYVQMQK.[G] | 1828.13 | 388-403 | 16 | -0.087 | 5.66 | 1.9 | 930.4402 | 1859.873 | 1859.888 | -7.89 | -0.00733 | 27.1931 |
| Transcription-repair-coupling factor OS=Streptococcus thermophilus OX=1308 GN=mfd PE=3 SV=1 | A0A8A3UUR5 | 1 | 1 | [R].FENHAVEVDELSR.[F] | 1544.64 | 686-698 | 13 | -0.708 | 4.4 | 2.75 | 515.5864 | 1544.745 | 1544.734 | 6.85 | 0.00353 | 16.1186 |
| Transposase OS=Lactobacillus sp. OX=1591 GN=DUD28_09900 PE=4 SV=1 | A0A3R8GD16 | 3 | 1 | [K].RQMSQATINVMMDIFR.[N] | 1941.31 | 51-66 | 16 | -0.062 | 9.6 | 3.37 | 658.3202 | 1972.946 | 1972.94 | 2.98 | 0.00196 | 17.2687 |
| Two component transcriptional regulator, winged helix family OS=Olsenella uli (strain ATCC 49627 / DSM 7084 / CIP 109912 / JCM 12494 / NCIMB 702895 / VPI D76D-27C) OX=633147 GN=Olsu_1229 PE=4 SV=1 | E1QW32 | 10 | 1 | [R].RVGGASGSTLACGGLSLDVARSR.[A] | 2190.46 | 118-140 | 23 | 0.209 | 10.26 | 2.13 | 1124.079 | 2247.151 | 2247.162 | -5.03 | -0.00566 | 25.2164 |
| Tyrosine-protein phosphatase OS=Limosilactobacillus gastricus PS3 OX=1144300 GN=PS3_19798 PE=3 SV=1 | H4GK04 | 9 | 1 | [R].AANLMVNAGQGTIMASDAHVMEK.[R] | 2359.72 | 181-203 | 23 | 0.196 | 5.32 | 2.06 | 1204.062 | 2407.117 | 2407.105 | 4.86 | 0.00585 | 26.2353 |
| Uncharacterized protein OS=Apilactobacillus apinorum OX=1218495 GN=RZ74_12160 PE=4 SV=1 | A0A0N0CNW4 | 1 | 1 | [R].EAGNNAIDAATNADGINSAYAAGK.[T] | 2279.36 | 859-882 | 24 | -0.375 | 4.03 | 2.64 | 570.5189 | 2279.054 | 2279.053 | 0.07 | 0.00004 | 15.4951 |
| Uncharacterized protein OS=Companilactobacillus ginsenosidimutans OX=1007676 GN=ABM34_10140 PE=4 SV=1 | A0A0H4QHG3 | 4 | 1 | [K].EVSRTTVQGADK.[S] | 1290.4 | 103-114 | 12 | -0.942 | 6.17 | 2.19 | 645.8327 | 1290.658 | 1290.665 | -5.18 | -0.00335 | 29.1341 |
| Uncharacterized protein OS=Lacticaseibacillus paracasei OX=1597 GN=FAM6012_02866 PE=4 SV=1 | A0A8B3GP59 | 18 | 1 | [K].GRMYQVSIDEIEYQPHFIMVYASEDAR.[D] | 3248.63 | 4-30 | 27 | -0.448 | 4.5 | 2.77 | 1083.184 | 3247.538 | 3247.519 | 6.08 | 0.00658 | 27.3557 |
| Uncharacterized protein OS=Lactobacillus iners LactinV 03V1-b OX=879298 GN=HMPREF9212_1023 PE=4 SV=1 | E1NPT1 | 2 | 1 | [K].DILAELDK.[L] | 916.04 | 280-287 | 8 | -0.062 | 4.03 | 2.2 | 458.7528 | 916.4983 | 916.4986 | -0.34 | -0.00016 | 21.5932 |
| Uncharacterized protein OS=Lactobacillus sp. OX=1591 GN=DCE17_07660 PE=4 SV=1 | A0A3C1XQ18 | 13 | 1 | [K].RLMEAFSVVYGGLHSKEDLENNNK.[M] | 2751.07 | 71-94 | 24 | -0.725 | 5.53 | 1.94 | 1383.687 | 2766.367 | 2766.352 | 5.58 | 0.00772 | 27.9356 |
| Uncharacterized protein OS=Limosilactobacillus reuteri OX=1598 GN=BHL82_02065 PE=4 SV=1 | A0A0U5JIG1 | 10 | 1 | [R].YPTISGHVTVARYTSSIQEADR.[F] | 2451.68 | 160-181 | 22 | -0.491 | 6.75 | 2.58 | 817.752 | 2451.241 | 2451.226 | 6.13 | 0.00501 | 26.5893 |
| Uncharacterized protein OS=Loigolactobacillus bifermentans OX=1607 GN=LB003_09770 PE=4 SV=1 | A0A5Q2P3H8 | 3 | 1 | [K].TQNTQLFNDFERMR.[V] | 1799.98 | 157-170 | 14 | -1.436 | 5.74 | 2.07 | 900.4253 | 1799.843 | 1799.849 | -3.35 | -0.00302 | 27.6824 |
| WxL domain-containing protein OS=Carnobacterium divergens OX=2748 GN=CKN67_05615 PE=4 SV=1 | A0A4R9CIE2 | 7 | 1 | [K].EKSSFNNVYDFLTTPDTSDR.[I] | 2336.45 | 157-176 | 20 | -1.16 | 4.23 | 2.47 | 779.364 | 2336.077 | 2336.068 | 4.11 | 0.0032 | 26.1479 |
| YSIRK-type signal peptide-containing protein (Fragment) OS=Ligilactobacillus salivarius OX=1624 GN=FYL25_08270 PE=4 SV=1 | A0A6N9ISV4 | 7 | 1 | [K].AASNQATDLDGNMGNLKESVADADTTK.[A] | 2737.89 | 383-409 | 27 | -0.781 | 4.04 | 2.42 | 913.0992 | 2737.283 | 2737.258 | 9.09 | 0.0083 | 18.6358 |
| YSIRK-type signal peptide-containing protein (Fragment) OS=Limosilactobacillus reuteri OX=1598 GN=GIX77_09685 PE=4 SV=1 | A0A7X2G5K4 | 1 | 1 | [K].FNNVTSPTINGYHADK.[T] | 1777.91 | 1606-1621 | 16 | -0.831 | 6.74 | 2.3 | 593.2836 | 1777.836 | 1777.85 | -8.01 | -0.00474 | 16.4914 |
| **Pepsin+Pancreatin** | | | | | | | | | | | | | | | | |
| Glycyl-radical enzyme activating protein family protein OS=Lactobacillus ultunensis DSM 16047 OX=525365 GN=pflC PE=3 SV=1 | C2ELS7 | 7 | 1 | [K].KETISYWVTVDDVMKEVEK.[D] | 2299.62 | 62-80 | 19 | -0.547 | 4.51 | 3.42 | 767.0632 | 2299.175 | 2299.153 | 9.79 | 0.00751 | 24.8344 |
| Putative peptidoglycan binding domain protein OS=Lentilactobacillus sunkii OX=481719 GN=LASUN_10840 PE=4 SV=1 | A0A1E7XD24 | 5 | 1 | [K].NLGRSHSVTMNLSHYAVGTGYGIK.[G] | 2562.89 | 305-328 | 24 | -0.233 | 9.7 | 2.58 | 854.7636 | 2562.276 | 2562.288 | -4.71 | -0.00402 | 26.9381 |
| Endonuclease MutS2 OS=Lactococcus sp. S-13 OX=2507158 GN=mutS2 PE=3 SV=1 | A0A4Q7DQF9 | 2 | 1 | [K].EQFLPALTTAQGQK.[E] | 1531.73 | 16-29 | 14 | -0.521 | 6.1 | 2.24 | 766.4047 | 1531.802 | 1531.811 | -6.16 | -0.00472 | 30.679 |
| Glycolate oxidase OS=Lactobacillus kullabergensis OX=1218493 GN=glcD PE=4 SV=1 | A0A0F4L9V8 | 6 | 1 | [R].GNSTGLMGANLAVDGGISLDMIKMNK.[V] | 2608.04 | 74-99 | 26 | 0.165 | 5.96 | 3.62 | 875.0991 | 2623.283 | 2623.289 | -2.36 | -0.00206 | 24.7434 |
| Single-stranded-DNA-specific exonuclease RecJ OS=Secundilactobacillus mixtipabuli OX=1435342 GN=recJ PE=3 SV=1 | A0A1Z5ICQ8 | 3 | 1 | [R].LDDAAPAVELLTTLDEERAEK.[L] | 2299.52 | 291-311 | 21 | -0.443 | 3.95 | 3.55 | 767.0632 | 2299.175 | 2299.166 | 3.82 | 0.00293 | 24.8344 |
| X-Pro dipeptidyl-peptidase OS=Apilactobacillus kunkeei OX=148814 GN=HW41_05550 PE=3 SV=1 | A0A0C2VVQ9 | 3 | 1 | [R].ENGLVMAPGGFQGEDADVLAAETYSR.[K] | 2697.91 | 408-433 | 26 | -0.273 | 3.83 | 3.14 | 899.7595 | 2697.264 | 2697.246 | 6.65 | 0.00598 | 23.7651 |
| N6_N4_Mtase domain-containing protein OS=Lactobacillus helveticus OX=1587 GN=BDKNPLJD_01994 PE=3 SV=1 | A0A2X0Q3Q1 | 4 | 1 | [R].NQSYNPQTRPNLYYPLYVDPNTK.[K] | 2786.05 | 288-310 | 23 | -1.517 | 8.38 | 3.07 | 929.1166 | 2785.335 | 2785.358 | -8.2 | -0.00761 | 24.6326 |
| Tannase OS=Lentilactobacillus curieae OX=1138822 GN=PL11_005860 PE=4 SV=1 | A0A1S6QIR3 | 4 | 1 | [K].SGTWTKELSNDMAKQYASYLNK.[L] | 2535.81 | 285-306 | 22 | -1.082 | 8.11 | 3.02 | 845.7415 | 2535.21 | 2535.218 | -3.32 | -0.00281 | 23.8676 |
| DUF1906 domain-containing protein OS=Lactobacillus casei OX=1582 GN=F9B82_06220 PE=4 SV=1 | A0A6L3UVX1 | 3 | 1 | [R].EALQHELGITDIGEGFGPATR.[S] | 2211.42 | 42-62 | 21 | -0.324 | 4.4 | 2.99 | 737.7065 | 2211.105 | 2211.104 | 0.39 | 0.00029 | 24.2145 |
| Uncharacterized protein OS=Limosilactobacillus reuteri OX=1598 GN=BHL82_02065 PE=4 SV=1 | A0A0U5JIG1 | 10 | 1 | [R].YPTISGHVTVARYTSSIQEADR.[F] | 2451.68 | 160-181 | 22 | -0.491 | 6.75 | 2.79 | 817.7517 | 2451.24 | 2451.226 | 5.76 | 0.0047 | 26.5092 |
| Probable 2-(5''-triphosphoribosyl)-3'-dephosphocoenzyme-A synthase OS=Lacticaseibacillus paracasei NRIC 0644 OX=1435038 GN=citG PE=3 SV=1 | A0A0C9PLK6 | 8 | 1 | [R].TIGVKAEKAMMTATVGVNTHR.[G] | 2215.61 | 76-96 | 21 | 0.01 | 9.99 | 2.72 | 749.7217 | 2247.151 | 2247.158 | -3.44 | -0.00258 | 25.1833 |
| Voltage gated chloride channel OS=Limosilactobacillus mucosae OX=97478 GN=SAMN05216430_1276 PE=4 SV=1 | A0A1H0F063 | 10 | 1 | [-].MGAAVGQGLAEKCFKSSHHETK.[V] | 2316.64 | 1-22 | 22 | -0.523 | 7.98 | 2.71 | 791.7239 | 2373.157 | 2373.144 | 5.67 | 0.00448 | 24.0382 |
| DUF3991 domain-containing protein OS=Ligilactobacillus salivarius ACS-116-V-Col5a OX=768728 GN=HMPREF9269_0303 PE=4 SV=1 | A0A828BXJ9 | 6 | 1 | [K].GIGGNLYQFMKEYLGMESTDILK.[E] | 2608.02 | 61-83 | 23 | -0.174 | 4.68 | 2.69 | 875.0976 | 2623.278 | 2623.278 | 0 | 0 | 24.5341 |
| DEAD/DEAH box helicase family protein OS=Lactobacillus panisapium OX=2012495 GN=GYM71_05080 PE=4 SV=1 | A0A8G0AED3 | 1 | 1 | [K].VTDLDLTAEVVK.[S] | 1302.49 | 335-346 | 12 | 0.517 | 4.03 | 2.68 | 651.8608 | 1302.714 | 1302.715 | -0.55 | -0.00036 | 28.8314 |
| 4-hydroxy-tetrahydrodipicolinate reductase OS=Lentilactobacillus parabuchneri OX=152331 GN=dapB PE=3 SV=1 | A0A1X1FED6 | 12 | 1 | [K].KNDDFELVGAYNPGIDTVHLQGMGMGDQVK.[L] | 3249.62 | 23-52 | 30 | -0.500 | 4.35 | 2.58 | 1088.844 | 3264.516 | 3264.53 | -4.18 | -0.00455 | 26.9947 |
| NADH oxidase OS=Lactiplantibacillus garii OX=2306423 GN=D1831_01465 PE=4 SV=1 | A0A3R8J9T3 | 6 | 1 | [R].IDAHAHTIMAQDLQTKEMVHDHYDK.[L] | 2948.28 | 82-106 | 25 | -0.864 | 5.6 | 2.56 | 983.1348 | 2947.39 | 2947.383 | 2.45 | 0.0024 | 24.4515 |
| Glyco_trans_2-like domain-containing protein OS=Lactobacillus sp. UMNPBX9 OX=2042038 GN=CP360_00190 PE=4 SV=1 | A0A2A7QSB4 | 5 | 1 | [K].IDVNEFSKTSKTTEEK.[K] | 1856.02 | 125-140 | 16 | -1.337 | 4.87 | 2.56 | 619.3191 | 1855.943 | 1855.928 | 7.74 | 0.00479 | 26.7909 |
| Uncharacterized protein OS=Schleiferilactobacillus shenzhenensis LY-73 OX=1231336 GN=L248_0838 PE=4 SV=1 | U4TRL3 | 8 | 1 | [R].WRYDLIGMETGSMGTYPFAYR.[I] | 2514.86 | 88-108 | 21 | -0.433 | 6.07 | 2.5 | 849.3924 | 2546.163 | 2546.148 | 5.79 | 0.00491 | 22.002 |
| Peptide ABC transporter substrate-binding protein OS=Lactobacillus helveticus OX=1587 GN=BCM45_03255 PE=4 SV=1 | A0A1B2IQ47 | 3 | 1 | [R].YTASLQDIVGLTDYHDGKSK.[T] | 2211.41 | 153-172 | 20 | -0.665 | 5.3 | 2.42 | 1106.055 | 2211.102 | 2211.093 | 4.31 | 0.00477 | 24.1693 |
| Uncharacterized protein OS=Finegoldia magna ACS-171-V-Col3 OX=768713 GN=HMPREF9261_1337 PE=4 SV=1 | D9PRR0 | 4 | 1 | [K].DNLTLIK.[N] | 815.97 | 29-35 | 7 | 0.071 | 5.84 | 2.42 | 408.7445 | 816.4818 | 816.4825 | -0.91 | -0.00037 | 20.2052 |
| Uncharacterized protein OS=Lactobacillus sp. UMNPBX2 OX=2042045 GN=CP353_06680 PE=4 SV=1 | A0A2A7RBN6 | 1 | 1 | [R].NSGNPQNTKGWNANGSTDILMVTK.[H] | 2547.78 | 854-877 | 24 | -0.979 | 8.59 | 2.41 | 849.7416 | 2547.21 | 2547.226 | -5.99 | -0.00509 | 26.1682 |
| Pbp2B2 protein OS=Lactobacillus selangorensis OX=81857 GN=IV38_GL000595 PE=3 SV=1 | A0A0R2GAG7 | 3 | 1 | [K].SVESTSNNGKKGQVESVTMPK.[V] | 2207.44 | 566-586 | 21 | -1.033 | 8.22 | 2.35 | 741.7058 | 2223.103 | 2223.092 | 4.74 | 0.00351 | 25.5109 |
| IS5 family transposase OS=Limosilactobacillus fermentum OX=1613 GN=E2P75_00775 PE=4 SV=1 | A0A4Z0CI89 | 8 | 1 | [R].RYDLYDVFNAILYSLTIGCQWR.[KE] | 2710.1 | 31-52 | 22 | 0.091 | 5.95 | 2.32 | 922.7927 | 2766.364 | 2766.371 | -2.61 | -0.0024 | 27.8877 |
| Uncharacterized protein OS=Lactobacillus bombicola OX=1505723 GN=DS834_03935 PE=4 SV=1 | A0A417ZLS8 | 6 | 1 | [K].EKNEELFYQLGNNPRCFVK.[A] | 2328.63 | 274-292 | 19 | -1.063 | 6.31 | 2.3 | 795.7246 | 2385.159 | 2385.166 | -2.72 | -0.00217 | 26.5142 |
| Degv family protein OS=Lactobacillus hamsteri DSM 5661 = JCM 6256 OX=1423754 GN=FC39_GL001000 PE=4 SV=1 | A0A0R1YBE9 | 4 | 1 | [R].AEGYEVLAAAK.[D] | 1121.26 | 126-136 | 11 | 0.236 | 4.53 | 2.29 | 561.2958 | 1121.584 | 1121.584 | 0.5 | 0.00028 | 19.2792 |
| 3-dehydro-L-gulonate-6-phosphate decarboxylase OS=Leuconostoc pseudomesenteroides OX=33968 GN=AMBR_MGDJBKAP_01368 PE=4 SV=1 | A0A370AEG6 | 4 | 1 | [K].KFISMGFK.[V] | 957.2 | 154-161 | 8 | 0.375 | 10 | 2.28 | 479.2608 | 957.5144 | 957.5226 | -8.61 | -0.00412 | 26.958 |
| Adsorption protein OS=Lactobacillus equicursoris DSM 19284 = JCM 14600 = CIP 110162 OX=1293597 GN=FC20_GL001195 PE=4 SV=1 | A0A0R1LY98 | 0 | 1 | [K].NNISTVK.[Q] | 774.87 | 799-805 | 7 | -0.529 | 8.75 | 2.26 | 388.2186 | 775.43 | 775.4308 | -1.11 | -0.00043 | 15.9697 |
| IS1380 family transposase OS=Liquorilactobacillus nagelii OX=82688 GN=BSQ50_10910 PE=4 SV=1 | A0A3S6R2W8 | 12 | 1 | [K].SASSTFNANAAR.[M] | 1196.24 | 17-28 | 12 | -0.383 | 9.47 | 2.21 | 598.786 | 1196.565 | 1196.565 | -0.58 | -0.00035 | 17.4121 |
| Uncharacterized protein OS=Lactobacillus iners LactinV 03V1-b OX=879298 GN=HMPREF9212_1023 PE=4 SV=1 | E1NPT1 | 2 | 1 | [K].DILAELDK.[L] | 916.04 | 280-287 | 8 | -0.062 | 4.03 | 2.2 | 458.753 | 916.4987 | 916.4986 | 0.13 | 0.00006 | 21.509 |
| DEAD-box ATP-dependent RNA helicase CshA OS=Carnobacterium divergens OX=2748 GN=cshA PE=3 SV=1 | A0A5F0ML74 | 2 | 1 | [K].SGGNRGKATDNR.[R] | 1232.28 | 518-529 | 12 | -2.025 | 10.83 | 2.19 | 616.8029 | 1232.599 | 1232.609 | -8.5 | -0.00524 | 16.2784 |
| D-alanine/D-serine/glycine permease OS=Limosilactobacillus mucosae OX=97478 GN=LX03_09555 PE=4 SV=1 | A0A099YA13 | 2 | 1 | [K].DPNNDIPK.[A] | 911.97 | 230-237 | 8 | -2.075 | 4.21 | 2.18 | 456.7247 | 912.4421 | 912.4421 | -0.08 | -0.00004 | 16.3064 |
| Uncharacterized protein OS=Companilactobacillus ginsenosidimutans OX=1007676 GN=ABM34_10140 PE=4 SV=1 | A0A0H4QHG3 | 4 | 1 | [K].EVSRTTVQGADK.[S] | 1290.4 | 103-114 | 12 | -0.942 | 6.17 | 2.17 | 645.833 | 1290.659 | 1290.665 | -4.71 | -0.00304 | 29.1162 |
| PhageMin_Tail domain-containing protein OS=Limosilactobacillus mucosae LM1 OX=1130798 GN=LBLM1_10880 PE=4 SV=1 | A0A0D4CNN7 | 0 | 1 | [K].VSDNQGLMNVAR.[W] | 1303.46 | 893-904 | 12 | -0.317 | 5.81 | 2.17 | 660.3254 | 1319.643 | 1319.637 | 4.74 | 0.00313 | 19.7474 |
| Peptidase_C39_2 domain-containing protein OS=Secundilactobacillus pentosiphilus OX=1714682 GN=IWT140_01257 PE=4 SV=1 | A0A1Z5IPX9 | 1 | 1 | [K].LDLAEIK.[QK] | 800.95 | 464-470 | 7 | 0.429 | 4.37 | 2.17 | 401.2391 | 801.4709 | 801.4717 | -0.89 | -0.00036 | 20.7217 |
| Formamidopyrimidine-DNA glycosylase OS=Lactococcus sp. S-13 OX=2507158 GN=mutM PE=3 SV=1 | A0A4Q7DT98 | 5 | 1 | [R].TYSALGEPGRMQEK.[L] | 1566.75 | 222-235 | 14 | -1.186 | 5.81 | 2.15 | 783.8827 | 1566.758 | 1566.758 | 0.02 | 0.00002 | 23.1341 |
| DUF3284 domain-containing protein OS=Lactobacillus xujianguonis OX=2495899 GN=EJK17_06445 PE=4 SV=1 | A0A437SUN2 | 9 | 1 | [K].RMADNVYAHLAN.[-] | 1374.54 | 125-136 | 12 | -0.35 | 6.74 | 2.15 | 695.8276 | 1390.648 | 1390.653 | -3.75 | -0.00261 | 19.6393 |
| LysR family transcriptional regulator OS=Lactobacillus sp. OX=1591 GN=DUD28_07140 PE=3 SV=1 | A0A3R8GGH8 | 4 | 1 | [K].QGRSVTLTEYGR.[I] | 1366.5 | 51-62 | 12 | -1.025 | 8.75 | 2.13 | 683.8524 | 1366.697 | 1366.707 | -7.26 | -0.00496 | 23.8429 |
| Uncharacterized protein OS=Secundilactobacillus folii OX=2678357 GN=GM612_05755 PE=4 SV=1 | A0A7X3C316 | 3 | 1 | [K].DLLIDAQDVQK.[E] | 1257.41 | 154-164 | 11 | -0.300 | 3.93 | 2.09 | 629.3335 | 1257.66 | 1257.669 | -6.99 | -0.0044 | 27.4287 |
| Histone acetyltransferase OS=Lactobacillus kalixensis DSM 16043 OX=1423763 GN=FC46_GL000217 PE=4 SV=1 | A0A0R1UAD4 | 6 | 1 | [KR].LSLNVDKENPR.[A] | 1284.43 | 148-158 | 11 | -1.182 | 6.07 | 2.07 | 642.8471 | 1284.687 | 1284.691 | -2.98 | -0.00191 | 17.714 |
| Two component transcriptional regulator, winged helix family OS=Olsenella uli (strain ATCC 49627 / DSM 7084 / CIP 109912 / JCM 12494 / NCIMB 702895 / VPI D76D-27C) OX=633147 GN=Olsu_1229 PE=4 SV=1 | E1QW32 | 10 | 1 | [R].RVGGASGSTLACGGLSLDVARSR.[A] | 2190.46 | 118-140 | 23 | 0.209 | 10.26 | 2.07 | 1124.08 | 2247.154 | 2247.162 | -3.84 | -0.00431 | 25.1733 |
| Uncharacterized protein OS=Levilactobacillus brevis OX=1580 GN=AZI11_13720 PE=4 SV=1 | A0A1W6NKI9 | 2 | 1 | [K].QSASLIYQLDDASK.[K] | 1538.67 | 436-449 | 14 | -0.421 | 4.21 | 2.06 | 769.8832 | 1538.759 | 1538.77 | -6.89 | -0.0053 | 22.8827 |
| Uncharacterized protein OS=Lactobacillus porci OX=2012477 GN=FYJ62_02200 PE=4 SV=1 | A0A6A8MBH9 | 2 | 1 | [K].AVAVPGLAR.[D] | 853.03 | 19-27 | 9 | 1.233 | 9.79 | 2.05 | 427.2661 | 853.5248 | 853.5254 | -0.69 | -0.00029 | 18.2603 |
| Helix-turn-helix domain-containing protein OS=Lactobacillus johnsonii OX=33959 GN=FEE39_09455 PE=4 SV=1 | A0A6P1YC23 | 34 | 1 | [-].MVSTKGEQLSMDLMLKKPQDR.[L] | 2435.9 | 1-21 | 21 | -0.795 | 8.25 | 2.05 | 1226.125 | 2451.242 | 2451.24 | 0.54 | 0.00067 | 26.5067 |
| Phage infection protein OS=Lacticaseibacillus paracasei subsp. paracasei 8700:2 OX=537973 GN=LBPG_03029 PE=4 SV=1 | A0A806KR01 | 2 | 1 | [-].MTNGLGTLQTK.[T] | 1163.35 | 1-11 | 11 | -0.391 | 8.50 | 2.05 | 590.31 | 1179.613 | 1179.604 | 7.56 | 0.00446 | 17.172 |
| Membrane protein OS=Dellaglioa algida OX=105612 GN=LABALGLTS371_07070 PE=4 SV=1 | A0A5C6MAX7 | 1 | 1 | [K].KMDLAELK.[F] | 947.16 | 376-383 | 8 | -0.438 | 6.07 | 2.04 | 482.2658 | 963.5243 | 963.5179 | 6.64 | 0.0032 | 20.9374 |
| Uncharacterized protein OS=Fructilactobacillus florum 8D OX=1221538 GN=B808_42 PE=4 SV=1 | W9EG57 | 5 | 1 | [R].DGSISIVDHGTEIAR.[E] | 1569.69 | 86-100 | 15 | -0.12 | 4.54 | 2.02 | 785.3969 | 1569.787 | 1569.787 | -0.11 | -0.00009 | 23.7137 |
| Phosphoadenosine phosphosulfate reductase family protein OS=Lactobacillus sp. 3B(2020) OX=2695882 GN=GTO83_03035 PE=4 SV=1 | A0A7L5UGI0 | 8 | 1 | [K].TQAQITDTVLISFSMGK.[D] | 1840.12 | 9-25 | 17 | 0.294 | 5.50 | 2.01 | 928.4757 | 1855.944 | 1855.947 | -1.54 | -0.00143 | 26.967 |
| LPXTG cell wall anchor domain-containing protein OS=Lactobacillus casei OX=1582 GN=KTT66_03595 PE=4 SV=1 | A0A8F4VDF7 | 1 | 1 | [R].VGNTSNSDKNRVDTNTHSGNK.[T] | 2245.31 | 2951-2971 | 21 | -1.757 | 8.57 | 2 | 1123.035 | 2245.064 | 2245.055 | 3.71 | 0.00417 | 26.356 |
| Uncharacterized protein OS=Lacticaseibacillus paracasei NRIC 0644 OX=1435038 GN=LC0644_2404 PE=4 SV=1 | A0A0C9P0F1 | 4 | 1 | [R].QVDIPAMLVATR.[A] | 1313.58 | 108-119 | 12 | 0.700 | 5.84 | 2 | 665.3669 | 1329.727 | 1329.72 | 5.35 | 0.00356 | 28.8088 |
| DNA/RNA non-specific endonuclease OS=Lacticaseibacillus rhamnosus OX=47715 GN=HWN39_14440 PE=4 SV=1 | A0A7Y7UK16 | 6 | 1 | [K].TNSAISSLDGAIADKR.[E] | 1618.76 | 48-63 | 16 | -0.263 | 5.63 | 1.99 | 809.9194 | 1618.831 | 1618.839 | -4.95 | -0.00401 | 30.5668 |
| Uncharacterized protein OS=Lactobacillus helveticus H9 OX=767456 GN=LBH_1357 PE=4 SV=1 | W5XNW4 | 2 | 1 | [R].KATGDNK.[I] | 732.79 | 145-151 | 7 | -2.014 | 8.59 | 1.99 | 367.1919 | 733.3766 | 733.3839 | -9.98 | -0.00366 | 21.4602 |
| AAA family ATPase OS=Lactobacillus helveticus OX=1587 GN=GDZ32_00590 PE=3 SV=1 | A0A6A7JZ50 | 4 | 1 | [K].ENGLGNYPVALTDDAR.[S] | 1704.81 | 166-181 | 16 | -0.688 | 4.03 | 1.98 | 852.9132 | 1704.819 | 1704.819 | 0.23 | 0.00019 | 21.8674 |
| Uncharacterized protein OS=Companilactobacillus kimchii OX=2801452 GN=LKACC12383_00911 PE=4 SV=1 | A0A210PBG8 | 2 | 1 | [K].ESGDYLGKWR.[N] | 1210.31 | 330-339 | 10 | -1.54 | 6.17 | 1.96 | 605.796 | 1210.585 | 1210.585 | -0.39 | -0.00023 | 18.2292 |
| Threonine--tRNA ligase OS=Loigolactobacillus bifermentans OX=1607 GN=thrS PE=3 SV=1 | A0A5Q2P4P4 | 2 | 1 | [R].MLKAGLRVEVDER.[N] | 1515.79 | 569-581 | 13 | -0.315 | 5.94 | 1.96 | 766.4221 | 1531.837 | 1531.826 | 7.02 | 0.00538 | 27.0278 |
| Uncharacterized protein OS=Ligilactobacillus ruminis SPM0211 OX=1040964 GN=LRU_01458 PE=4 SV=1 | F7R190 | 7 | 1 | [K].NVDDASNDVTFSALRK.[M] | 1751.87 | 227-242 | 16 | -0.600 | 4.43 | 1.96 | 876.4398 | 1751.872 | 1751.856 | 9.35 | 0.00819 | 25.9868 |
| Phage protein OS=Lacticaseibacillus paracasei subsp. paracasei Lpp41 OX=1256208 GN=Lpp41_07998 PE=4 SV=1 | A0A829H6C4 | 20 | 1 | [K].DRPIDPAIMWKQYQEK.[G] | 2018.32 | 63-78 | 16 | -1.406 | 6.12 | 1.96 | 1009.503 | 2017.999 | 2018.016 | -8.77 | -0.00885 | 26.8355 |
| Pseudouridine synthase OS=Limosilactobacillus fermentum OX=1613 GN=BUW47_10890 PE=3 SV=1 | A0A1L7GXK3 | 5 | 1 | [K].KMMEAVGHPVLK.[L] | 1339.68 | 196-207 | 12 | 0.108 | 8.60 | 1.95 | 678.3566 | 1355.706 | 1355.717 | -8.51 | -0.00577 | 16.7581 |
| Uncharacterized protein OS=Companilactobacillus bobalius OX=2801451 GN=LKACC16343_01106 PE=4 SV=1 | A0A202FCK6 | 3 | 1 | [R].TNEDPYTIDVES.[-] | 1382.4 | 453-464 | 12 | -1.158 | 3.43 | 1.95 | 691.8065 | 1382.606 | 1382.596 | 7.12 | 0.00492 | 17.7575 |
| XRE family transcriptional regulator OS=Lactobacillus helveticus OX=1587 GN=DM470_02245 PE=4 SV=1 | A0A2V4EM93 | 5 | 1 | [K].EQIFDLPSIDLNK.[L] | 1531.73 | 140-152 | 13 | -0.338 | 4.03 | 1.94 | 766.4029 | 1531.799 | 1531.8 | -1.14 | -0.00087 | 30.4685 |
| Mucus binding protein OS=Lactobacillus gallinarum DSM 10532 = JCM 2011 OX=1423748 GN=FC37_GL000910 PE=4 SV=1 | A0A0R1NS66 | 5 | 1 | [K].YIGAGSDTPKEITDHTTFTR.[H] | 2210.39 | 98-117 | 20 | -0.825 | 5.38 | 1.94 | 1105.546 | 2210.086 | 2210.072 | 5.93 | 0.00655 | 27.9435 |
| Uncharacterized protein OS=Liquorilactobacillus nagelii OX=82688 GN=BSQ50_09350 PE=4 SV=1 | A0A3Q8CZU5 | 2 | 1 | [K].DIIDIDALQK.[M] | 1143.30 | 25-34 | 10 | 0.120 | 3.93 | 1.93 | 572.317 | 1143.627 | 1143.626 | 1.03 | 0.00059 | 23.1104 |
| 3-hexulose-6-phosphate synthase OS=Levilactobacillus brevis KB290 OX=1001583 GN=LVISKB_0449 PE=4 SV=1 | M5ABD3 | 6 | 1 | [K].QLFIDMMEITANK.[T] | 1553.85 | 104-116 | 13 | 0.200 | 4.37 | 1.92 | 777.3941 | 1553.781 | 1553.77 | 6.9 | 0.00536 | 30.5439 |
| Single-stranded-DNA-specific exonuclease RecJ OS=Lactiplantibacillus pentosus OX=1589 GN=BB562_07065 PE=3 SV=1 | A0A2K9I0M7 | 2 | 1 | [R].IMKQVEQYMGAQAK.[A] | 1624.93 | 597-610 | 14 | -0.529 | 8.50 | 1.9 | 820.9058 | 1640.804 | 1640.813 | -5.62 | -0.00461 | 30.471 |
| Tyrosine-protein phosphatase OS=Limosilactobacillus gastricus PS3 OX=1144300 GN=PS3_19798 PE=3 SV=1 | H4GK04 | 9 | 1 | [R].AANLMVNAGQGTIMASDAHVMEK.[R] | 2359.72 | 181-203 | 23 | 0.196 | 5.32 | 1.9 | 1204.06 | 2407.113 | 2407.105 | 3.44 | 0.00414 | 26.1707 |
| Glycosyl transferase OS=Lentilactobacillus curieae OX=1138822 GN=PL11_005170 PE=4 SV=1 | A0A1S6QID2 | 1 | 1 | [K].GEHNIVMTYATPGFK.[L] | 1664.90 | 987-1001 | 15 | -0.267 | 6.75 | 1.9 | 832.9045 | 1664.802 | 1664.81 | -4.98 | -0.00415 | 21.538 |
| **Pepsin+Pancreatin+Trypsin** | | | | | | | | | | | | | | | | |
| Alpha-glucosidase OS=Lacticaseibacillus paracasei NRIC 0644 OX=1435038 GN=LC0644_1090 PE=4 SV=1 | A0A0C9PNH5 | 42 | 1 | [K].ADFPNTFILGEAASANVNLAVDYTSQHNK.[L] | 3108.329 | 247-275 | 29 | -0.21724 | 4.53 | 3.12 | 757.0112 | 2269.019 | 2269.026 | -3.19 | -0.00241 | 23.1185 |
| Uncharacterized protein OS=Lactobacillus phage JNU_P4 OX=2686383 PE=4 SV=1 | A0A6M3BEH3 | 23 | 1 | [R].DNAQGDPRDQNQGASQTNSNSRGTENGIK.[N] | 3060.039 | 172-200 | 29 | -1.97586 | 4.68 | 2.02 | 1135.015 | 2269.022 | 2269.026 | -1.95 | -0.00221 | 23.1914 |
| DUF1542 domain-containing protein (Fragment) OS=Limosilactobacillus reuteri OX=1598 GN=GIX74_08215 PE=4 SV=1 | A0A6A8DU33 | 21 | 1 | [K].TDEINNVSNLSTDEKQDLINQASEAAK.[N] | 2948.068 | 1693-1719 | 27 | -1.01481 | 4.05 | 2.3 | 735.6902 | 2205.056 | 2205.067 | -4.98 | -0.00366 | 23.9912 |
| Elongation factor Ts OS=Lactobacillus helsingborgensis OX=1218494 GN=tsf PE=3 SV=1 | A0A0F4M331 | 16 | 1 | [K].NIAMHVAAINPEYLDKDSVPKADFDR.[Q] | 2930.251 | 178-203 | 26 | -0.46923 | 4.75 | 2.83 | 962.7979 | 2886.379 | 2886.403 | -8.16 | -0.00785 | 26.7515 |
| Thymidylate kinase OS=Dellaglioa algida OX=105612 GN=tmk PE=3 SV=1 | A0A5C6M9V9 | 15 | 1 | [R].EPGGNPISEAVRDVVLNQDYPEMDKR.[T] | 2929.178 | 40-65 | 26 | -1.05769 | 4.29 | 2.71 | 702.9925 | 2106.963 | 2106.948 | 7.3 | 0.00513 | 23.4724 |
| Uncharacterized protein OS=Lactobacillus bombicola OX=1505723 GN=SAMN04487792_0050 PE=4 SV=1 | A0A1I1R8J3 | 14 | 1 | [R].EAAPFALDMNYYEIDGNTPSAFYGIK.[N] | 2898.157 | 186-211 | 26 | -0.26154 | 4.05 | 2.44 | 914.4577 | 2741.359 | 2741.349 | 3.37 | 0.00308 | 26.8855 |
| ABC transporter permease OS=Lactobacillus sp. ESL0246 OX=2069359 GN=F5ESL0246_01095 PE=4 SV=1 | A0A3M0LVQ3 | 13 | 1 | [K].TEIGLAMRATGDNSEMSAANGINTQAMK.[I] | 2883.196 | 158-185 | 28 | -0.36786 | 4.67 | 2.33 | 821.0838 | 2461.237 | 2461.26 | -9.52 | -0.00781 | 25.1226 |
| Intracellular maltogenic amylase OS=Lacticaseibacillus paracasei OX=1597 GN=FAM18157_00472 PE=4 SV=1 | A0A422M7P6 | 13 | 1 | [K].DTESARIDTDNYFRVPYFHEIDR.[V] | 2860.01 | 111-133 | 23 | -1.17391 | 4.51 | 2.11 | 1383.687 | 2766.366 | 2766.352 | 5.32 | 0.00735 | 27.8501 |
| N-acetyltransferase domain-containing protein OS=Limosilactobacillus reuteri OX=1598 GN=DKZ23_08355 PE=4 SV=1 | A0A317GHA5 | 12 | 1 | [R].LFTSCGYKEWGRLPQISEINGQMR.[S] | 2814.202 | 83-106 | 24 | -0.57917 | 8.19 | 1.98 | 1075.536 | 2150.064 | 2150.059 | 2.6 | 0.0028 | 24.8546 |
| Single-stranded-DNA-specific exonuclease RecJ OS=Weissella confusa OX=1583 GN=recJ PE=3 SV=1 | A0A4Z0S1E3 | 12 | 1 | [K].HYEQLAPTIKAPAEAVYWEDAFSR.[T] | 2793.049 | 591-614 | 24 | -0.54167 | 4.83 | 3.02 | 767.0626 | 2299.173 | 2299.168 | 2.36 | 0.00181 | 24.9974 |
| DUF2075 domain-containing protein OS=Companilactobacillus farciminis KCTC 3681 = DSM 20184 OX=936140 GN=LF20184_02180 PE=4 SV=1 | A0A2D1KZG8 | 12 | 1 | [K].LNSSEDSYYDSTKMLFENITRTR.[K] | 2770.977 | 436-458 | 23 | -1.08696 | 4.78 | 2.38 | 976.8115 | 2928.42 | 2928.416 | 1.44 | 0.00141 | 27.7147 |
| Replicative DNA helicase OS=Limosilactobacillus coleohominis 101-4-CHN OX=575594 GN=dnaB PE=3 SV=1 | C7XXJ0 | 11 | 1 | [R].ESGSIEQDADIVSFLYRDDYYQR.[E] | 2769.881 | 382-404 | 23 | -0.96087 | 4.05 | 1.91 | 1019.461 | 2037.916 | 2037.921 | -2.46 | -0.00251 | 25.0499 |
| Multidrug transporter OS=Lactiplantibacillus pentosus OX=1589 GN=BB562_03635 PE=3 SV=1 | A0A2K9I2J6 | 11 | 1 | [R].MIQAMGVGISAPVFQAIMSSVYPPEK.[R] | 2752.275 | 117-142 | 26 | 0.626923 | 5.75 | 2.15 | 1168.543 | 2336.079 | 2336.072 | 3.07 | 0.00359 | 26.0225 |
| Uncharacterized protein OS=Lactobacillus sp. OX=1591 GN=DCE17_07660 PE=4 SV=1 | A0A3C1XQ18 | 11 | 1 | [K].RLMEAFSVVYGGLHSKEDLENNNK.[M] | 2751.035 | 71-94 | 24 | -0.725 | 5.53 | 2.87 | 522.9105 | 1566.717 | 1566.722 | -2.98 | -0.00156 | 17.8487 |
| AAA family ATPase OS=Ligilactobacillus salivarius OX=1624 GN=B6U61_06020 PE=3 SV=1 | A0A1V9QHH2 | 11 | 1 | [R].TVVTVNGADVVLNQCIMHNFSNTNK.[K] | 2719.058 | 99-123 | 25 | 0.136 | 6.4 | 2.33 | 1020.466 | 3059.384 | 3059.376 | 2.7 | 0.00275 | 25.4912 |
| IS5 family transposase OS=Limosilactobacillus fermentum OX=1613 GN=E2P75_00775 PE=4 SV=1 | A0A4Z0CI89 | 10 | 1 | [R].RYDLYDVFNAILYSLTIGCQWR.[KE] | 2710.069 | 31-52 | 22 | 0.090909 | 5.95 | 1.91 | 928.4749 | 1855.943 | 1855.933 | 5.01 | 0.00465 | 27.1519 |
| Fructose-1,6-bisphosphatase class 3 OS=Lactiplantibacillus plantarum OX=1590 GN=fbp PE=3 SV=1 | A0A0R2G5W1 | 10 | 1 | [K].MTCATIQHLTVDHIHIVGDIYDR.[G] | 2652.013 | 190-212 | 23 | 0.195652 | 5.69 | 2.29 | 1124.08 | 2247.152 | 2247.162 | -4.6 | -0.00517 | 25.1297 |
| MFS transporter OS=Levilactobacillus zymae OX=267363 GN=LZ395_09465 PE=4 SV=1 | A0A5P8PWR3 | 10 | 1 | [R].FLAGVGAGGEYGVGITLIAESFDHDK.[I] | 2623.866 | 114-139 | 26 | 0.373077 | 4.308 | 2.51 | 821.0811 | 2461.229 | 2461.211 | 7.31 | 0.00599 | 24.6886 |
| Uncharacterized protein OS=Lactobacillus sp. SL9-6 OX=2604480 GN=FXE12_11680 PE=4 SV=1 | A0A5D0JP06 | 10 | 1 | [-].MKMSVLIITVMATFSLASCANHNK.[T] | 2611.175 | ‘1-24 | 24 | 0.816667 | 9.305 | 1.93 | 928.4756 | 1855.944 | 1855.936 | 4.38 | 0.00407 | 26.749 |
| Glycolate oxidase OS=Lactobacillus kullabergensis OX=1218493 GN=glcD PE=4 SV=1 | A0A0F4L9V8 | 9 | 1 | [R].GNSTGLMGANLAVDGGISLDMIKMNK.[V] | 2608.02 | 74-99 | 26 | 0.165385 | 5.95 | 2.91 | 817.7524 | 2451.243 | 2451.226 | 6.73 | 0.0055 | 26.4799 |
| Beta sliding clamp OS=Carnobacterium divergens OX=2748 GN=dnaN PE=3 SV=1 | A0A2R8A496 | 9 | 1 | [R].TLDERHENIEMMITENQVLFK.[T] | 2590.925 | 216-236 | 21 | -0.64762 | 4.57 | 2.4 | 972.1246 | 2914.359 | 2914.334 | 8.72 | 0.00847 | 25.6757 |
| Putative peptidoglycan binding domain protein OS=Lentilactobacillus sunkii OX=481719 GN=LASUN_10840 PE=4 SV=1 | A0A1E7XD24 | 9 | 1 | [K].NLGRSHSVTMNLSHYAVGTGYGIK.[G] | 2562.857 | 305-328 | 24 | -0.23333 | 9.7 | 3.13 | 707.9988 | 2121.982 | 2121.988 | -2.66 | -0.00188 | 23.8412 |
| Type III restriction-modification system endonuclease OS=Limosilactobacillus mucosae OX=97478 GN=LM011_07025 PE=4 SV=1 | A0A7L9VRL7 | 9 | 1 | [K].QEFVDELPADLLGVYSADAVADPK.[Y] | 2562.778 | 852-875 | 24 | -0.00417 | 4.05 | 2.45 | 832.0393 | 2494.103 | 2494.094 | 3.78 | 0.00314 | 27.6803 |
| Tannase OS=Lentilactobacillus curieae OX=1138822 GN=PL11_005860 PE=4 SV=1 | A0A1S6QIR3 | 9 | 1 | [K].SGTWTKELSNDMAKQYASYLNK.[L] | 2535.782 | 285-306 | 22 | -1.08182 | 8.105 | 3.11 | 749.0258 | 2245.063 | 2245.078 | -6.74 | -0.00504 | 26.3205 |
| Holliday junction resolvase RecU OS=Lactobacillus sp. OX=1591 GN=recU PE=3 SV=1 | A0A425X6T5 | 9 | 1 | [R].FDNRGMSLEEEINESNQFYR.[A] | 2478.605 | 30-49 | 20 | -1.4 | 4.25 | 3.18 | 803.043 | 2407.115 | 2407.105 | 3.94 | 0.00316 | 26.1265 |
| Uncharacterized protein OS=Apilactobacillus kunkeei OX=148814 GN=RZ55_05770 PE=4 SV=1 | A0A0N0CR90 | 9 | 1 | [K].LISDTSDKINGDHVPGQSVNDQK.[T] | 2467.601 | 1797-1819 | 23 | -1.01304 | 4.55 | 2.47 | 1204.062 | 2407.116 | 2407.105 | 4.46 | 0.00536 | 26.125 |
| L-2-hydroxyisocaproate dehydrogenase OS=Limosilactobacillus fermentum OX=1613 GN=E2P74_06425 PE=4 SV=1 | A0A6D1XS29 | 8 | 1 | [R].FLGQPDTFAQLAGNAQAIQEWR.[S] | 2461.685 | 78-99 | 22 | -0.36818 | 4.37 | 2.35 | 982.4869 | 2945.446 | 2945.446 | 0.03 | 0.00003 | 27.7223 |
| Guanylate kinase OS=Lactobacillus gasseri (strain ATCC 33323 / DSM 20243 / BCRC 14619 / CIP 102991 / JCM 1131 / KCTC 3163 / NCIMB 11718 / NCTC 13722 / AM63) OX=324831 GN=LGAS_1700 PE=4 SV=1 | A0A805ZRN7 | 8 | 1 | [R].DLHLSDELAKEAHVLNNDNWK.[E] | 2461.641 | 170-190 | 21 | -0.99524 | 4.79 | 1.9 | 869.9251 | 1738.843 | 1738.854 | -6.52 | -0.00567 | 22.9048 |
| Peptidase OS=Limosilactobacillus reuteri OX=1598 GN=GIX81_03325 PE=4 SV=1 | A0A6L5P206 | 8 | 1 | [K].NRLSNGMLGISAYIYPGQKDVR.[V] | 2452.786 | 1189-1210 | 22 | -0.42727 | 9.69 | 2.78 | 922.7927 | 2766.364 | 2766.371 | -2.61 | -0.0024 | 27.845 |
| Uncharacterized protein OS=Limosilactobacillus reuteri OX=1598 GN=BHL82_02065 PE=4 SV=1 | A0A0U5JIG1 | 8 | 1 | [R].YPTISGHVTVARYTSSIQEADR.[F] | 2451.646 | 160-181 | 22 | -0.49091 | 6.75 | 3.07 | 695.0078 | 2083.009 | 2083.012 | -1.8 | -0.00125 | 26.4384 |
| Transcriptional regulator, Rrf2 family OS=Lactiplantibacillus pentosus KCA1 OX=1136177 GN=KCA1_0194 PE=4 SV=1 | I8R942 | 8 | 1 | [K].DHKSLKSNVMSQILEVSDSSLK.[K] | 2445.744 | 22-43 | 22 | -0.5 | 6.75 | 2.39 | 766.4038 | 1531.8 | 1531.815 | -9.55 | -0.00732 | 30.6364 |
| Threonylcarbamoyl-AMP synthase OS=Secundilactobacillus pentosiphilus OX=1714682 GN=SUA5 PE=3 SV=1 | A0A1Z5IQT1 | 8 | 1 | [K].YKHYAPSAQVEIVDHPEDFK.[A] | 2373.574 | 227-246 | 20 | -0.95 | 5.31 | 2.05 | 689.8516 | 1378.696 | 1378.696 | -0.11 | -0.00007 | 17.5247 |
| Tyrosine-protein phosphatase OS=Limosilactobacillus gastricus PS3 OX=1144300 GN=PS3_19798 PE=3 SV=1 | H4GK04 | 7 | 1 | [R].AANLMVNAGQGTIMASDAHVMEK.[R] | 2359.701 | 181-203 | 23 | 0.195652 | 5.32 | 2.95 | 695.0078 | 2083.009 | 2082.991 | 8.35 | 0.0058 | 26.4384 |
| DNA polymerase III PolC-type OS=Companilactobacillus alimentarius DSM 20249 OX=1423720 GN=polC PE=3 SV=1 | A0A2K9HPP1 | 7 | 1 | [K].QAMKDANVPDWYIDSCLKIK.[Y] | 2338.7 | 1237-1256 | 20 | -0.44 | 6.03 | 2.16 | 1042.007 | 2083.007 | 2082.991 | 7.71 | 0.00803 | 26.4158 |
| 2-deoxyuridine 5-triphosphate nucleotidohydrolase OS=Lactobacillus sp. OX=1591 GN=DUD35_03755 PE=4 SV=1 | A0A425XPG0 | 7 | 1 | [K].FGLVDFGYTEQEIQDAFNDK.[N] | 2336.464 | 148-167 | 20 | -0.6 | 4.05 | 2.07 | 611.3275 | 1221.648 | 1221.637 | 8.31 | 0.00507 | 28.0449 |
| Chromosome partition protein Smc OS=Lacticaseibacillus fabifermentans T30PCM01 OX=1400520 GN=smc PE=3 SV=1 | W6T9J7 | 7 | 1 | [K].QADAQDQLADLNDAIEDFTDK.[Q] | 2336.378 | 890-910 | 21 | -1 | 4.05 | 2.53 | 745.0115 | 2233.02 | 2233.03 | -4.55 | -0.00339 | 16.261 |
| Rqc2 homolog RqcH OS=Lactobacillus melliventris OX=1218507 GN=pavA PE=3 SV=1 | A0A0F4LG79 | 7 | 1 | [K].TMHSDPVDFASQIGGLDRDDR.[Q] | 2332.462 | 185-205 | 21 | -0.9 | 4.27 | 2.58 | 565.3017 | 1693.891 | 1693.887 | 2.22 | 0.00125 | 26.9149 |
| Sugar ABC transporter substrate-binding protein OS=Lactobacillus rodentium OX=947835 GN=ugpB PE=4 SV=1 | A0A2Z6T5Y4 | 7 | 1 | [K].YTMQQGTDIYMFNQGSADQK.[A] | 2326.517 | 306-325 | 20 | -1.055 | 4.207 | 3.73 | 821.0811 | 2461.229 | 2461.226 | 1.11 | 0.00091 | 24.9302 |
| Glycyl-radical enzyme activating protein family protein OS=Lactobacillus ultunensis DSM 16047 OX=525365 GN=pflC PE=3 SV=1 | C2ELS7 | 7 | 1 | [K].KETISYWVTVDDVMKEVEK.[D] | 2299.595 | 62-80 | 19 | -0.54737 | 4.513 | 3.88 | 767.0631 | 2299.175 | 2299.153 | 9.56 | 0.00732 | 24.8132 |
| Uncharacterized protein OS=Lactobacillus kitasatonis DSM 16761 = JCM 1039 OX=1423767 GN=FC59_GL000438 PE=4 SV=1 | A0A0R1VGQ9 | 7 | 1 | [K].DFTIVPHAGGNVKSLTSAQKDN.[-] | 2299.495 | 157-178 | 22 | -0.49091 | 6.746 | 2.3 | 1123.036 | 2245.064 | 2245.063 | 0.45 | 0.0005 | 26.284 |
| Phage protein, major head protein OS=Lactobacillus hominis DSM 23910 = CRBIP 24.179 OX=1423758 GN=BN55_04265 PE=4 SV=1 | I7KHQ1 | 7 | 1 | [R].QQMQIDITNTGAGSFETDTTK.[L] | 2286.429 | 339-359 | 21 | -0.85714 | 4.05 | 3.67 | 725.3466 | 2174.025 | 2174.026 | -0.16 | -0.00012 | 26.1956 |
| Uncharacterized protein OS=Lentilactobacillus buchneri subsp. silagei CD034 OX=1071400 GN=LBUCD034_0027 PE=4 SV=1 | J9W4F8 | 6 | 1 | [R].ITNDSQTSQSRRGMGAADPGGY.[-] | 2269.365 | 31-52 | 22 | -1.12727 | 5.96 | 2.05 | 970.9716 | 1940.936 | 1940.939 | -1.67 | -0.00162 | 25.9894 |
| DNA topoisomerase OS=Lactobacillus jensenii OX=109790 GN=F6H94_06710 PE=3 SV=1 | A0A5N1IB96 | 6 | 1 | [K].FWGFSFDVNKTEFPDDGTR.[T] | 2265.391 | 671-689 | 19 | -0.84211 | 4.22 | 1.91 | 803.4408 | 1605.874 | 1605.86 | 9.23 | 0.00741 | 26.8389 |
| DAK2 domain-containing protein OS=Lactobacillus xujianguonis OX=2495899 GN=EJK17_01320 PE=4 SV=1 | A0A437SXP1 | 6 | 1 | [R].VAAQEGAEKANDTDDVEEVMK.[A] | 2249.366 | 139-159 | 21 | -0.89048 | 4.05 | 1.96 | 876.439 | 1751.871 | 1751.856 | 8.44 | 0.00739 | 25.9459 |
| Uncharacterized protein OS=Lactobacillus sp. UMNPBX5 OX=2042042 GN=CP356_04505 PE=4 SV=1 | A0A2A7R0Z8 | 6 | 1 | [R].QVTKKANGEIVYGEWDQPGK.[M] | 2247.462 | 443-462 | 20 | -1.185 | 6.17 | 2.37 | 673.3366 | 2017.995 | 2018.008 | -6.15 | -0.00413 | 26.8314 |
| Excinuclease ABC OS=Lentilactobacillus farraginis DSM 18382 = JCM 14108 OX=1423743 GN=JCM14108_2834 PE=4 SV=1 | X0PBU3 | 6 | 1 | [R].GNMIFLDEPTAGLHMQDIDK.[L] | 2245.531 | 201-220 | 20 | -0.31 | 4.21 | 3.38 | 648.9758 | 1944.913 | 1944.907 | 2.81 | 0.00182 | 23.3218 |
| SEC10/PgrA surface exclusion domain-containing protein OS=Lactobacillus equicursoris OX=420645 GN=FYJ61_02160 PE=4 SV=1 | A0A844FLM9 | 6 | 1 | [K].EVTDGSEVVTVVNTDEEPSTK.[S] | 2235.313 | 1167-1187 | 21 | -0.65714 | 4.05 | 1.91 | 590.3093 | 1179.611 | 1179.612 | -0.24 | -0.00014 | 16.8808 |
| TIGR00266 family protein OS=Lactobacillus delbrueckii subsp. lactis OX=29397 GN=yfhL PE=4 SV=1 | A0A061CN99 | 6 | 1 | [K].MKSQSFSKAAFGGTGGFYVMK.[T] | 2229.576 | 119-139 | 21 | -0.05714 | 10 | 3.13 | 875.0982 | 2623.28 | 2623.304 | -9.07 | -0.00793 | 24.5242 |
| Fumarylacetoacetate hydrolase domain-containing protein 2A OS=Companilactobacillus kimchii OX=2801452 GN=hpaG PE=4 SV=1 | A0A210P6L1 | 6 | 1 | [K].QNVANEHIAGYMVGQDLSDR.[Q] | 2217.375 | 128-147 | 20 | -0.645 | 4.53 | 2 | 1087.515 | 2174.022 | 2174.036 | -6.33 | -0.00688 | 26.1654 |
| Peptide ABC transporter substrate-binding protein OS=Lactobacillus helveticus OX=1587 GN=BCM45_03255 PE=4 SV=1 | A0A1B2IQ47 | 6 | 1 | [R].YTASLQDIVGLTDYHDGKSK.[T] | 2211.384 | 153-172 | 20 | -0.665 | 5.29 | 2.46 | 791.7244 | 2373.159 | 2373.151 | 3.18 | 0.00252 | 24.0796 |
| Co-chaperonin GroES OS=Lactobacillus gigeriorum DSM 23908 = CRBIP 24.85 OX=1423751 GN=groES PE=3 SV=1 | I7J3Q8 | 6 | 1 | [K].EKPTEGEVVAVGEGAFATNGDK.[L] | 2205.335 | 32-53 | 22 | -0.57273 | 4.25 | 2.33 | 1009.503 | 2017.998 | 2018.012 | -7.26 | -0.00733 | 26.9951 |
| Two component transcriptional regulator, winged helix family OS=Olsenella uli (strain ATCC 49627 / DSM 7084 / CIP 109912 / JCM 12494 / NCIMB 702895 / VPI D76D-27C) OX=633147 GN=Olsu_1229 PE=4 SV=1 | E1QW32 | 6 | 1 | [R].RVGGASGSTLACGGLSLDVARSR.[A] | 2190.441 | 118-140 | 23 | 0.208696 | 10.26 | 2.16 | 809.9188 | 1618.83 | 1618.839 | -5.71 | -0.00462 | 30.5815 |
| Rpn family recombination-promoting nuclease/putative transposase OS=Limosilactobacillus reuteri OX=1598 GN=G5T19_08355 PE=4 SV=1 | A0A6M1BJA5 | 5 | 1 | [R].DGQTDILFNIPSSNHDVSSK.[M] | 2174.281 | 152-171 | 20 | -0.71 | 4.41 | 2.14 | 809.9207 | 1618.834 | 1618.839 | -3.3 | -0.00267 | 30.3791 |
| Uncharacterized protein OS=Lentilactobacillus otakiensis DSM 19908 = JCM 15040 OX=1423780 GN=LOT_1135 PE=4 SV=1 | S4NHE3 | 5 | 1 | [K].KGLMNSPKFPPQDPDYTSK.[L] | 2150.41 | 73-91 | 19 | -1.45263 | 8.43 | 3.66 | 875.0989 | 2623.282 | 2623.289 | -2.57 | -0.00225 | 24.7299 |
| tRNA dimethylallyltransferase OS=Lactobacillus xujianguonis OX=2495899 GN=miaA PE=3 SV=1 | A0A437ST23 | 5 | 1 | [K].MMEQGLLEEARFVYDHR.[Q] | 2124.399 | 212-228 | 17 | -0.65882 | 4.83 | 3.64 | 875.0989 | 2623.282 | 2623.289 | -2.57 | -0.00225 | 24.7299 |
| Uncharacterized protein OS=Schleiferilactobacillus harbinensis OX=304207 GN=LHA01_28880 PE=4 SV=1 | A0A510TZC8 | 5 | 1 | [K].FVSYSTHDLSSGQFNGTFK.[-] | 2122.249 | 183-201 | 19 | -0.41579 | 6.74 | 2.53 | 869.4237 | 2606.257 | 2606.259 | -0.9 | -0.00078 | 25.0599 |
| Alpha/beta hydrolase OS=Limosilactobacillus reuteri OX=1598 GN=LR3_03435 PE=4 SV=1 | A0A073JQR9 | 5 | 1 | [K].VTIMGDSAGGGLAAGFCEYLGK.[KR] | 2117.401 | 174-195 | 22 | 0.581818 | 4.37 | 2.63 | 708.6753 | 2124.011 | 2124 | 5.28 | 0.00374 | 23.0742 |
| Uncharacterized protein OS=Amylolactobacillus amylophilus DSM 20533 = JCM 1125 OX=1423721 GN=FD40_GL000863 PE=4 SV=1 | A0A0R1YIZ0 | 5 | 1 | [R].VDSEDHAHAPKNTQWEDK.[R] | 2107.154 | 70-87 | 18 | -1.93889 | 4.79 | 2.71 | 966.4488 | 2897.332 | 2897.334 | -0.7 | -0.00067 | 25.6395 |
| CRISPR-associated protein, Cse1 family OS=Amylolactobacillus amylophilus DSM 20533 = JCM 1125 OX=1423721 GN=FD40_GL001342 PE=4 SV=1 | A0A0R1YG74 | 5 | 1 | [R].QELLDSWNQTFTSGSFSR.[K] | 2103.204 | 99-116 | 18 | -0.81667 | 4.37 | 2.19 | 540.2668 | 1079.526 | 1079.537 | -9.7 | -0.00524 | 21.6855 |
| Uncharacterized protein OS=Lactobacillus sp. HMSC068F07 OX=1739365 GN=HMPREF2861_07225 PE=3 SV=1 | A0A1F1J103 | 5 | 1 | [K].ERERQQEIDEDTQEAAR.[H] | 2103.122 | 349-365 | 17 | -2.41765 | 4.25 | 2.98 | 928.4676 | 2783.388 | 2783.382 | 2.36 | 0.00219 | 27.8796 |
| N6_Mtase domain-containing protein OS=Lentilactobacillus sunkii OX=481719 GN=LASUN_01190 PE=4 SV=1 | A0A1E7XJ37 | 5 | 1 | [R].NISTFDSIIGQSDLMAGSAR.[K] | 2083.279 | 175-194 | 20 | 0.12 | 4.2 | 3.09 | 762.6927 | 2286.064 | 2286.055 | 3.53 | 0.00269 | 23.0893 |
| NAD-dependent protein deacetylase OS=Lacticaseibacillus paracasei OX=1597 GN=cobB PE=4 SV=1 | K0MY36 | 5 | 1 | [K].HVTFMTGAGVSTASGIPDYR.[S] | 2067.282 | 28-47 | 20 | 0.11 | 6.74 | 2.9 | 1036.506 | 3107.503 | 3107.507 | -1.35 | -0.0014 | 27.5621 |
| Cation transport ATPase OS=Lacticaseibacillus paracasei subsp. paracasei Lpp122 OX=1256218 GN=Lpp122_0593 PE=4 SV=1 | A0A8E0M3T6 | 5 | 1 | [R].SEPSDILNFLELAHNTSR.[K] | 2043.194 | 609-626 | 18 | -0.52222 | 4.64 | 2.72 | 854.7634 | 2562.276 | 2562.288 | -4.92 | -0.00421 | 26.8804 |
| Uncharacterized protein OS=Lactobacillus sp. LL6 OX=2596827 GN=FOD82_07495 PE=4 SV=1 | A0A556UDV2 | 5 | 1 | [K].QDQSVDGKEVDSNTVSSDK.[Q] | 2038.042 | 138-156 | 19 | -1.44737 | 4.05 | 2.27 | 1281.644 | 2562.281 | 2562.288 | -2.68 | -0.00343 | 26.8931 |
| Energy-coupling factor transporter ATP-binding protein EcfA1 OS=Lentilactobacillus parabuchneri OX=152331 GN=FAM23169_01670 PE=3 SV=1 | A0A1X1FDK1 | 5 | 1 | [K].GEIIEENKPENIFGETAK.[L] | 2018.181 | 214-231 | 18 | -0.96111 | 4.325 | 2.03 | 783.8818 | 1566.756 | 1566.758 | -1.07 | -0.00084 | 23.1411 |
| ATP-dependent helicase/nuclease subunit A OS=Ligilactobacillus equi DPC 6820 OX=1392007 GN=addA PE=3 SV=1 | V7HUB1 | 5 | 1 | [K].YYAGDFNDGQEDSDFNR.[L] | 2012.951 | 170-186 | 17 | -1.72353 | 4.05 | 2.43 | 678.3569 | 1355.707 | 1355.717 | -7.97 | -0.0054 | 16.6967 |
| Carboxypeptidase OS=Companilactobacillus farciminis KCTC 3681 = DSM 20184 OX=936140 GN=LF20184_09335 PE=4 SV=1 | A0A2D1KXQ5 | 5 | 1 | [K].EEILEDYLNAATFGANNK.[G] | 2012.134 | 194-211 | 18 | -0.58333 | 4.05 | 2.4 | 678.3569 | 1355.707 | 1355.717 | -7.97 | -0.0054 | 16.6967 |
| 6-phospho-beta-glucosidase OS=Companilactobacillus nuruki OX=1993540 GN=CBP76_02715 PE=3 SV=1 | A0A2N7AWD3 | 4 | 1 | [K].AERAMQASYWFADVQCK.[G] | 2004.249 | 255-271 | 17 | -0.4 | 6.104 | 2.62 | 923.7548 | 2769.25 | 2769.264 | -5.1 | -0.0047 | 28.8808 |
| ABC transporter, Glycine betaine/L-proline transporter ATPase component OS=Lactobacillus kullabergensis OX=1218493 GN=opuCa PE=3 SV=1 | A0A0F4LDS2 | 4 | 1 | [K].IDLGASLTDALTMMKEHR.[V] | 2002.316 | 269-286 | 18 | -0.07778 | 5.38 | 2.75 | 929.4299 | 2786.275 | 2786.294 | -6.65 | -0.00617 | 26.1341 |
| Alpha,alpha-phosphotrehalase OS=Lactiplantibacillus plantarum 2025 OX=1385856 GN=N876_0205610 PE=4 SV=1 | A0A837NM54 | 4 | 1 | [-].MYPASFKDANNDGIGDLR.[G] | 1984.15 | ‘1-18 | 18 | -0.76667 | 4.42 | 2.27 | 565.3143 | 1129.621 | 1129.621 | 0.09 | 0.00005 | 19.0786 |
| Uncharacterized protein OS=Lactobacillus gasseri (strain ATCC 33323 / DSM 20243 / BCRC 14619 / CIP 102991 / JCM 1131 / KCTC 3163 / NCIMB 11718 / NCTC 13722 / AM63) OX=324831 GN=LGAS_0384 PE=4 SV=1 | A0A805Z6T6 | 4 | 1 | [R].NSKKDHGQVNFECIITK.[H] | 1961.203 | 180-196 | 17 | -0.92941 | 8.18 | 2.43 | 781.3374 | 2341.998 | 2342.006 | -3.72 | -0.00291 | 18.2247 |
| Uncharacterized protein OS=Lactiplantibacillus mudanjiangensis OX=1296538 GN=MUDAN_MDHGFNIF_00784 PE=4 SV=1 | A0A660E348 | 4 | 1 | [K].EAVKHNGSHYIYLETAK.[G] | 1960.15 | 207-223 | 17 | -0.77059 | 7.01 | 2.4 | 673.3361 | 2017.994 | 2018.005 | -5.31 | -0.00357 | 27.027 |
| Uncharacterized protein OS=Limosilactobacillus reuteri OX=1598 GN=CBG21_07260 PE=4 SV=1 | A0A256VGU1 | 4 | 1 | [K].ATSQAEAPQSTTVNDQGVR.[T] | 1960.021 | 242-260 | 19 | -0.91579 | 4.37 | 2.32 | 673.3361 | 2017.994 | 2018.005 | -5.31 | -0.00357 | 27.027 |
| Gp58 domain-containing protein OS=Weissella confusa OX=1583 GN=C6P11_02715 PE=4 SV=1 | A0A4Z0S0J8 | 4 | 1 | [R].FESGDTVTNGYLEQSGLK.[L] | 1945.044 | 829-846 | 18 | -0.68333 | 4.13 | 2.02 | 818.4382 | 1635.869 | 1635.87 | -0.61 | -0.0005 | 27.5357 |
| Replication protein OS=Lactobacillus phage PM411 OX=2079298 PE=4 SV=1 | A0A2P0ZLB0 | 4 | 1 | [R].DGTVDVFTDLNIDYTEK.[S] | 1945.041 | 168-184 | 17 | -0.54118 | 4.05 | 1.91 | 755.8545 | 1510.702 | 1510.702 | -0.19 | -0.00014 | 15.8678 |
| Phage_lysozyme2 domain-containing protein OS=Lactiplantibacillus paraplantarum OX=60520 GN=LPA07_12800 PE=4 SV=1 | A0A512FJ33 | 4 | 1 | [K].HAKDQNSSVVDQANSQSK.[G] | 1942.994 | 1235-1252 | 18 | -1.48333 | 6.74 | 2.03 | 891.9584 | 1782.909 | 1782.901 | 4.89 | 0.00436 | 19.8482 |
| Uncharacterized protein OS=Companilactobacillus suantsaicola OX=2487723 GN=EGT49_07355 PE=4 SV=1 | A0A4Z0JMA0 | 4 | 1 | [R].GDHALGAGYQGLGVMGYDK.[A] | 1909.083 | 142-160 | 19 | -0.27895 | 5.21 | 2.41 | 653.9973 | 1959.977 | 1959.992 | -7.7 | -0.00503 | 27.5382 |
| Glutamine ABC transporter ATP-binding protein OS=Liquorilactobacillus hordei OX=468911 GN=glnQ PE=4 SV=1 | A0A3Q8CS05 | 4 | 1 | [R].QKMTMIIVTHEMGFAR.[Q] | 1893.301 | 185-200 | 16 | 0.19375 | 8.75 | 2.1 | 758.2971 | 1515.587 | 1515.587 | -0.04 | -0.00003 | 25.2493 |
| Restriction endonuclease subunit R OS=Ligilactobacillus salivarius OX=1624 GN=B7R82_10500 PE=4 SV=1 | A0A1Y0FAK2 | 4 | 1 | [K].DTITIDMNDSTVEHER.[S] | 1875.964 | 567-582 | 16 | -1.03125 | 4.1 | 2.09 | 758.297 | 1515.587 | 1515.587 | -0.2 | -0.00015 | 25.0162 |
| GRAM_POS_ANCHORING domain-containing protein OS=Limosilactobacillus reuteri OX=1598 GN=HF82_03830 PE=4 SV=1 | A0A073JNZ7 | 4 | 1 | [K].VTQGSINFAKSVAENYK.[N] | 1856.041 | 375-391 | 17 | -0.38235 | 8.46 | 2.08 | 683.8518 | 1366.696 | 1366.707 | -8.15 | -0.00557 | 23.8816 |
| Uncharacterized protein OS=Lactobacillus johnsonii ATCC 33200 OX=525330 GN=FC22_GL001157 PE=4 SV=1 | A0A0R1IHR4 | 4 | 1 | [K].TMNIIYPPLVEQMYK.[F] | 1840.21 | ‘3-17 | 15 | -0.00667 | 5.66 | 2.44 | 953.7771 | 2859.317 | 2859.333 | -5.78 | -0.0055 | 23.6197 |
| Uncharacterized protein OS=Ligilactobacillus murinus OX=1622 GN=CPQ89_04795 PE=4 SV=1 | A0A2Z4VY74 | 4 | 1 | [-].MEIKVSDFITAVTEEK.[F] | 1840.099 | ‘1-16 | 16 | -0.00625 | 4.4 | 2.34 | 645.8328 | 1290.658 | 1290.665 | -5.09 | -0.00328 | 29.0794 |
| Protein translocase subunit SecA OS=Lentilactobacillus hilgardii OX=1588 GN=secA PE=3 SV=1 | A0A6G9Q729 | 3 | 1 | [R].EVIYGERQEVIEENK.[D] | 1834.977 | 609-623 | 15 | -1.14667 | 4.32 | 2.06 | 645.8323 | 1290.657 | 1290.665 | -5.75 | -0.00371 | 29.2501 |
| RNA methyltransferase OS=Companilactobacillus heilongjiangensis OX=1074467 GN=JP39_05780 PE=4 SV=1 | A0A0K2LC81 | 3 | 1 | [K].DVVEITLDTTGESLYK.[R] | 1782.939 | 144-159 | 16 | -0.125 | 4.05 | 2.74 | 755.6849 | 2265.04 | 2265.019 | 9.43 | 0.00712 | 25.6707 |
| Beta-glucosidase OS=Companilactobacillus nuruki OX=1993540 GN=CBP76_07800 PE=3 SV=1 | A0A2N7ATM5 | 3 | 1 | [R].EATKLAMDATVDIDMK.[S] | 1752.016 | 280-295 | 16 | -0.09375 | 4.22 | 2.37 | 778.0209 | 2332.048 | 2332.062 | -6 | -0.00467 | 28.0651 |
| Uncharacterized protein OS=Ligilactobacillus ruminis SPM0211 OX=1040964 GN=LRU_01458 PE=4 SV=1 | F7R190 | 3 | 1 | [K].NVDDASNDVTFSALRK.[M] | 1751.848 | 227-242 | 16 | -0.6 | 4.42 | 2.05 | 1051.999 | 2102.99 | 2102.97 | 9.66 | 0.01016 | 25.8217 |
| Uncharacterized protein OS=Lactobacillus phage SAC12B OX=2510941 GN=SAC12B_0062 PE=4 SV=1 | A0A4Y5FFG3 | 3 | 1 | [K].LWMLAAEFAMTPVDK.[R] | 1723.064 | 69-83 | 15 | 0.646667 | 4.37 | 2.42 | 852.9136 | 1704.82 | 1704.819 | 0.73 | 0.00062 | 21.8657 |
| Transcription-repair-coupling factor OS=Streptococcus thermophilus OX=1308 GN=mfd PE=3 SV=1 | A0A8A3UUR5 | 3 | 1 | [K].TEVAMRAAFKAVNDGK.[Q] | 1707.948 | 648-663 | 16 | -0.225 | 8.25 | 3.07 | 845.7408 | 2535.208 | 2535.218 | -4.19 | -0.00354 | 23.8663 |
| AAA family ATPase OS=Lactobacillus helveticus OX=1587 GN=GDZ32_00590 PE=3 SV=1 | A0A6A7JZ50 | 3 | 1 | [K].ENGLGNYPVALTDDAR.[S] | 1704.792 | 166-181 | 16 | -0.6875 | 4.05 | 2.03 | 575.2982 | 1149.589 | 1149.59 | -0.61 | -0.00035 | 17.425 |
| YhfK OS=Apilactobacillus kunkeei OX=148814 GN=RZ78_00470 PE=4 SV=1 | A0A0P7K2K2 | 3 | 1 | [K].LSKTGDNVFAGVRSDK.[Q] | 1693.855 | 21-36 | 16 | -0.56875 | 8.58 | 1.9 | 1038.964 | 2076.921 | 2076.927 | -2.73 | -0.00283 | 17.0692 |
| Extracellular protein, peptide binding protein OppA-like protein [Lactobacillus plantarum JDM1] OS=Lactiplantibacillus mudanjiangensis OX=1296538 GN=MUDAN_MDHGFNIF_00110 PE=4 SV=1 | A0A660DUT6 | 3 | 1 | [K].TAMQDQAVTPLYEGR.[S] | 1679.849 | 504-518 | 15 | -0.64667 | 4.36 | 2.45 | 908.7813 | 2724.329 | 2724.323 | 2.26 | 0.00205 | 26.8704 |
| PDZ domain-containing protein OS=Lactobacillus sp. OX=1591 GN=DUD28_00010 PE=3 SV=1 | A0A3R8NHA7 | 3 | 1 | [K].YPSYAYLPMVSDQK.[T] | 1661.871 | 389-402 | 14 | -0.56429 | 5.83 | 1.92 | 833.8976 | 1666.788 | 1666.785 | 1.53 | 0.00127 | 21.5069 |
| Amino acid permease OS=Lactobacillus johnsonii OX=33959 GN=E6A54_06540 PE=4 SV=1 | A0A6B9HZ27 | 3 | 1 | [K].TANGGVSSWVNATMGAK.[W] | 1650.811 |  | 17 | -0.01765 | 8.41 | 2.13 | 770.3823 | 1539.757 | 1539.747 | 6.55 | 0.00504 | 20.7711 |
| Protein translocase subunit SecA OS=Ligilactobacillus equi DPC 6820 OX=1392007 GN=secA PE=3 SV=1 | V7HWV3 | 3 | 1 | [K].QLFEQAQMLEFEK.[V] | 1640.854 |  | 13 | -0.61538 | 4.25 | 2.18 | 1106.059 | 2211.11 | 2211.093 | 7.85 | 0.00868 | 24.1843 |
| DUF2974 domain-containing protein OS=Lactobacillus xujianguonis OX=2495899 GN=EJK17_02545 PE=4 SV=1 | A0A437SWN5 | 2 | 1 | [K].LTQKSYQELALGER.[I] | 1635.816 |  | 14 | -0.88571 | 6.14 | 2.34 | 661.9757 | 1983.913 | 1983.923 | -5.21 | -0.00344 | 17.2891 |
| Glycoside hydrolase family 65 protein OS=Lactobacillus sp. OX=1591 GN=DUD28_04990 PE=3 SV=1 | A0A425X6P8 | 2 | 1 | [K].YRGRQIDIDINQK.[T] | 1618.792 |  | 13 | -1.43077 | 8.58 | 2.21 | 539.7795 | 1078.552 | 1078.556 | -4 | -0.00216 | 21.7736 |
| DNA/RNA non-specific endonuclease OS=Lacticaseibacillus rhamnosus OX=47715 GN=HWN39_14440 PE=4 SV=1 | A0A7Y7UK16 | 2 | 1 | [K].TNSAISSLDGAIADKR.[E] | 1618.744 |  | 16 | -0.2625 | 5.62 | 2.59 | 701.6674 | 2102.988 | 2102.978 | 4.72 | 0.00331 | 25.8685 |
| Probable nicotinate-nucleotide adenylyltransferase OS=Lactobacillus pasteurii DSM 23907 = CRBIP 24.76 OX=1423790 GN=nadD PE=3 SV=1 | I7J0L5 | 2 | 1 | [K].TGTSIRYLVPEDVR.[K] | 1605.79 |  | 14 | -0.34286 | 5.73 | 2.32 | 931.4636 | 2792.376 | 2792.368 | 2.96 | 0.00276 | 26.2433 |
| ATP-dependent helicase/deoxyribonuclease subunit B OS=Levilactobacillus brevis OX=1580 GN=rexB PE=3 SV=1 | A0A3B8F5I7 | 2 | 1 | [R].QQSRTALQYSPYK.[S] | 1569.716 |  | 13 | -1.52308 | 9.69 | 2.17 | 458.2608 | 915.5143 | 915.5146 | -0.33 | -0.00015 | 20.0755 |
| Formamidopyrimidine-DNA glycosylase OS=Lactococcus sp. S-13 OX=2507158 GN=mutM PE=3 SV=1 | A0A4Q7DT98 | 2 | 1 | [R].TYSALGEPGRMQEK.[L] | 1566.734 |  | 14 | -1.18571 | 5.809 | 2 | 831.3939 | 1661.781 | 1661.788 | -4.46 | -0.0037 | 25.7392 |
| GatB/YqeY domain-containing protein OS=Schleiferilactobacillus oryzae JCM 18671 OX=1291743 GN=LOSG293_060480 PE=4 SV=1 | A0A081BHC6 | 2 | 1 | [K].QAIADTNAAGMGDFGK.[V] | 1566.691 |  | 16 | -0.2125 | 4.207 | 2.43 | 560.6091 | 1679.813 | 1679.806 | 4.11 | 0.0023 | 16.5924 |
| Uncharacterized protein OS=Levilactobacillus brevis OX=1580 GN=AZI11_13720 PE=4 SV=1 | A0A1W6NKI9 | 2 | 1 | [K].QSASLIYQLDDASK.[K] | 1538.654 |  | 14 | -0.42143 | 4.207 | 2.61 | 925.7878 | 2775.349 | 2775.355 | -2.33 | -0.00216 | 26.239 |
| Endonuclease MutS2 OS=Lactococcus sp. S-13 OX=2507158 GN=mutS2 PE=3 SV=1 | A0A4Q7DQF9 | 2 | 1 | [K].EQFLPALTTAQGQK.[E] | 1531.708 |  | 14 | -0.52143 | 6.1 | 2.76 | 681.6726 | 2043.003 | 2043.014 | -5.33 | -0.00363 | 23.8512 |
| Pyrimidine-nucleoside phosphorylase OS=Paucilactobacillus hokkaidonensis JCM 18461 OX=1291742 GN=LOOC260_118980 PE=3 SV=1 | A0A0A1GZI1 | 2 | 1 | [K].TGAGAFMKVEADAQK.[L] | 1523.709 |  | 15 | -0.24667 | 5.73 | 1.95 | 691.8065 | 1382.606 | 1382.596 | 7.12 | 0.00492 | 17.7513 |
| Threonine--tRNA ligase OS=Loigolactobacillus bifermentans OX=1607 GN=thrS PE=3 SV=1 | A0A5Q2P4P4 | 2 | 1 | [R].MIKAGLRVEVDER.[N] | 1515.777 |  | 13 | -0.26154 | 5.93 | 3.11 | 749.723 | 2247.155 | 2247.14 | 6.27 | 0.0047 | 25.1681 |
| PTS Gat IIA OS=Lactobacillus melliventris OX=1218507 GN=JF74_03340 PE=4 SV=1 | A0A0F4LJS8 | 2 | 1 | [K].MLQKVADIVQNEK.[L] | 1515.773 |  | 13 | -0.37692 | 5.82 | 2.79 | 854.7634 | 2562.276 | 2562.261 | 5.68 | 0.00485 | 26.8804 |
| Uncharacterized protein OS=Lactiplantibacillus plantarum OX=1590 GN=IYO1511_22410 PE=4 SV=1 | A0A6F9Z4D4 | 2 | 1 | [K].ENDEQFDDDFNK.[L] | 1515.447 |  | 12 | -2.48333 | 4.05 | 2.67 | 653.9818 | 1959.931 | 1959.937 | -3.03 | -0.00198 | 24.1286 |
| Uncharacterized protein OS=Latilactobacillus curvatus OX=28038 GN=C0W45_00540 PE=4 SV=1 | A0A8D4LFB7 | 2 | 1 | [K].AYDQVVAENTSEGK.[-] | 1510.558 |  | 14 | -0.9 | 4.13 | 2.18 | 572.3164 | 1143.625 | 1143.626 | -0.15 | -0.00008 | 23.1335 |
| Uncharacterized protein OS=Companilactobacillus bobalius OX=2801451 GN=LKACC16343_01106 PE=4 SV=1 | A0A202FCK6 | 2 | 1 | [R].TNEDPYTIDVES.[-] | 1382.383 |  | 12 | -1.15833 | 4.05 | 2.04 | 954.9471 | 1908.887 | 1908.891 | -2.09 | -0.00199 | 27.0346 |
| LMWPc domain-containing protein OS=Lactobacillus sp. ASF360 OX=97137 GN=C821_01083 PE=3 SV=1 | N1ZR76 | 2 | 1 | [R].ITGGDPDRKEYK.[L] | 1378.487 |  | 12 | -1.89167 | 6.12 | 2.09 | 1133.025 | 2265.043 | 2265.025 | 7.97 | 0.00902 | 25.6244 |
| LysR family transcriptional regulator OS=Lactobacillus sp. OX=1591 GN=DUD28_07140 PE=3 SV=1 | A0A3R8GGH8 | 2 | 1 | [K].QGRSVTLTEYGR.[I] | 1366.479 |  | 12 | -1.025 | 8.74 | 1.91 | 616.8025 | 1232.598 | 1232.609 | -9.19 | -0.00567 | 16.2682 |
| Pseudouridine synthase OS=Limosilactobacillus fermentum OX=1613 GN=BUW47_10890 PE=3 SV=1 | A0A1L7GXK3 | 2 | 1 | [K].KMMEAVGHPVLK.[L] | 1339.669 |  | 12 | 0.108333 | 8.59 | 1.98 | 876.4352 | 1751.863 | 1751.855 | 4.46 | 0.0039 | 26.3407 |
| DEAD/DEAH box helicase family protein OS=Lactobacillus panisapium OX=2012495 GN=GYM71_05080 PE=4 SV=1 | A0A8G0AED3 | 2 | 1 | [K].VTDLDLTAEVVK.[S] | 1302.47 |  | 12 | 0.516667 | 4.05 | 1.93 | 642.325 | 1283.643 | 1283.63 | 9.83 | 0.00631 | 17.6294 |
| Uncharacterized protein OS=Companilactobacillus ginsenosidimutans OX=1007676 GN=ABM34_10140 PE=4 SV=1 | A0A0H4QHG3 | 2 | 1 | [K].EVSRTTVQGADK.[S] | 1290.38 |  | 12 | -0.94167 | 6.16 | 2.13 | 501.2739 | 1001.541 | 1001.537 | 3.14 | 0.00157 | 27.5872 |
| Oligopeptide ABC transporter substrate-binding protein OS=Secundilactobacillus pentosiphilus OX=1714682 GN=oppA_3 PE=4 SV=1 | A0A1Z5IR73 | 2 | 1 | [R].TVEFVQSQMAK.[L] | 1267.451 |  | 11 | -0.09091 | 5.66 | 2.61 | 619.319 | 1855.943 | 1855.955 | -6.63 | -0.0041 | 26.754 |
| Cytidine deaminase OS=Lactobacillus sp. HMSC08B12 OX=1581136 GN=HMPREF3168_00495 PE=3 SV=1 | A0A1F1R2L8 | 2 | 1 | [R].TAIFSWVNAGR.[QT] | 1221.364 |  | 11 | 0.390909 | 9.41 | 2.19 | 769.8854 | 1538.763 | 1538.77 | -4.03 | -0.0031 | 22.891 |
| Uncharacterized protein OS=Lactobacillus casei OX=1582 GN=AUQ39_05025 PE=4 SV=1 | A0A1Q9MMI4 | 2 | 1 | [R].FSGITKERANG.[-] | 1179.283 |  | 11 | -0.78182 | 8.74 | 1.98 | 766.4222 | 1531.837 | 1531.826 | 7.26 | 0.00556 | 26.9911 |
| Glycoside hydrolase family 25 OS=Lactobacillus sp. UMNPBX5 OX=2042042 GN=CP356_07550 PE=3 SV=1 | A0A2A7QZ14 | 2 | 1 | [K].VSQGTGYVNPK.[Y] | 1149.254 |  | 11 | -0.7 | 8.56 | 1.98 | 766.4222 | 1531.837 | 1531.826 | 7.26 | 0.00556 | 26.9911 |
| Uncharacterized protein OS=Liquorilactobacillus nagelii OX=82688 GN=BSQ50_09350 PE=4 SV=1 | A0A3Q8CZU5 | 2 | 1 | [K].DIIDIDALQK.[M] | 1143.287 |  | 10 | 0.12 | 4.05 | 1.98 | 467.2376 | 933.4679 | 933.471 | -3.33 | -0.00156 | 18.9791 |
| 23S rRNA (Guanosine(2251)-2'-O)-methyltransferase RlmB OS=Levilactobacillus brevis OX=1580 GN=rlmB PE=4 SV=1 | A0A3B8F523 | 1 | 1 | [K].GASALIIGNEGK.[G] | 1129.264 |  | 12 | 0.291667 | 6.001 | 1.98 | 1006.49 | 2011.973 | 2011.961 | 6.01 | 0.00605 | 26.3193 |
| Dihydrofolate reductase OS=Lactobacillus psittaci DSM 15354 OX=1122152 GN=FC23_GL000609 PE=3 SV=1 | A0A0R1S829 | 1 | 1 | [K].KENFVADEK.[N] | 1079.161 |  | 9 | -1.44444 | 4.67 | 1.94 | 488.7404 | 976.4736 | 976.4694 | 4.24 | 0.00207 | 24.2657 |
| DEAD-box ATP-dependent RNA helicase CshA OS=Carnobacterium divergens OX=2748 GN=cshA PE=3 SV=1 | A0A5F0ML74 | 1 | 1 | [K].SGGNRGKATDNR.[R] | 1076.079 |  | 11 | -1.8 | 8.46 | 2.11 | 917.958 | 1834.909 | 1834.918 | -5.19 | -0.00476 | 30.8375 |
| Diacylglycerol kinase family lipid kinase OS=Lactobacillus iners OX=147802 GN=G6Z83_05225 PE=3 SV=1 | A0A6G7B9S7 | 1 | 1 | [R].KMDIGKAGDK.[Y] | 1062.241 |  | 10 | -1.13 | 8.49 | 2.18 | 946.4183 | 1891.829 | 1891.834 | -2.4 | -0.00227 | 27.2757 |
| Uncharacterized protein OS=Carnobacterium divergens OX=2748 GN=CKN77_01285 PE=4 SV=1 | A0A7I0FKP1 | 1 | 1 | [K].DNIIVKLSN.[-] | 1015.161 |  | 9 | 0.2 | 5.83 | 2.02 | 766.4027 | 1531.798 | 1531.811 | -8.71 | -0.00667 | 30.4655 |
| MFS transporter OS=Lacticaseibacillus rhamnosus OX=47715 GN=GFB59_04520 PE=4 SV=1 | A0A873ZFQ8 | 1 | 1 | [R].LTQGIAADGR.[L] | 1001.095 |  | 10 | -0.11 | 5.83 | 3 | 779.3621 | 2336.072 | 2336.052 | 8.21 | 0.00639 | 26.0241 |
| ATP-dependent protease ATPase subunit HslU OS=Ligilactobacillus agilis OX=1601 GN=hslU PE=3 SV=1 | A0A6F9Y2J8 | 1 | 1 | [R].SQATQQADK.[R] | 975.9996 |  | 9 | -1.75556 | 5.55 | 2.1 | 456.7246 | 912.4419 | 912.4421 | -0.28 | -0.00013 | 16.2562 |
| Membrane protein OS=Dellaglioa algida OX=105612 GN=LABALGLTS371_07070 PE=4 SV=1 | A0A5C6MAX7 | 1 | 1 | [K].KMDLAELK.[F] | 947.1504 |  | 8 | -0.4375 | 6.06 | 2.22 | 508.293 | 1015.579 | 1015.578 | 0.42 | 0.00021 | 18.2558 |
| 6-phospho-beta-glucosidase OS=Lactiplantibacillus plantarum OX=1590 GN=AVR83_07830 PE=3 SV=1 | A0A0G9FB16 | 1 | 1 | [K].DAMLIDGAK.[V] | 933.0807 |  | 9 | 0.277778 | 4.207 | 2.47 | 809.9381 | 1618.869 | 1618.866 | 1.76 | 0.00142 | 27.1178 |
| tRNA pseudouridine synthase A OS=Lactiplantibacillus mudanjiangensis OX=1296538 GN=truA PE=3 SV=1 | A0A660E2H1 | 1 | 1 | [K].DLQADLIK.[D] | 915.0423 |  | 8 | -0.0625 | 4.207 | 2.54 | 745.6912 | 2235.059 | 2235.051 | 3.52 | 0.00263 | 20.088 |
| D-alanine/D-serine/glycine permease OS=Limosilactobacillus mucosae OX=97478 GN=LX03_09555 PE=4 SV=1 | A0A099YA13 | 1 | 1 | [K].DPNNDIPK.[A] | 911.9556 |  | 8 | -2.075 | 4.2 | 1.91 | 820.9086 | 1640.81 | 1640.799 | 6.77 | 0.00556 | 30.4428 |
| Peptidase_C39_2 domain-containing protein OS=Secundilactobacillus pentosiphilus OX=1714682 GN=IWT140_01257 PE=4 SV=1 | A0A1Z5IPX9 | 1 | 1 | [K].LDLAEIK.[QK] | 800.9397 |  | 7 | 0.428571 | 4.37 | 2.59 | 1206.092 | 2411.177 | 2411.173 | 1.42 | 0.00171 | 26.2911 |
| LEA family epithelial adhesin OS=Limosilactobacillus reuteri OX=1598 GN=lea PE=4 SV=1 | A0A7H9EHI4 | 0 | 1 | [K].NNISISK.[E] | 774.8626 |  | 7 | -0.5 | 8.75 | 2.09 | 401.2391 | 801.4709 | 801.4717 | -0.89 | -0.00036 | 20.6643 |

**S1.2. Table: Selected peptides which passed physicochemical property and instability index screening**

| **Sequence** | **MW [Da]** | **No of AA** | **toxicity prediction** | **allergic prediction** | **Instability index** | **Antimicrobial** |
| --- | --- | --- | --- | --- | --- | --- |
| LSNRAAFFR | 1081.24 | 9 | non-toxin | non-allergen | stable | AMP |
| VTDLDLTAEVVK | 1302.49 | 10 | non-toxin | non-allergen | stable | AMP |
| SGGNRGKATDNR | 1232.28 | 12 | non-toxin | non-allergen | stable | NAMP |
| EVSRTTVQGADK | 1290.4 | 12 | non-toxin | non-allergen | stable | NAMP |
| YRGRQIDIDINQK | 1618.81 | 13 | non-toxin | non-allergen | stable | NAMP |
| MLQKVADIVQNEK | 1515.79 | 13 | non-toxin | non-allergen | stable | NAMP |
| YKTTGTVIHGEAR | 1432.6 | 13 | non-toxin | non-allergen | stable | NAMP |
| FENHAVEVDELSR | 1544.64 | 13 | non-toxin | non-allergen | stable | AMP |
| LGADATVPFDMTTK | 1466.67 | 14 | non-toxin | non-allergen | stable | AMP |
| TQNTQLFNDFERMR | 1799.98 | 14 | non-toxin | non-allergen | stable | NAMP |
| QMNLMAMTPMDVMNK | 1755.17 | 15 | non-toxin | non-allergen | stable | NAMP |
| FASIAPNVDTGEPTK | 1546.7 | 15 | non-toxin | non-allergen | stable | NAMP |
| KSSLVTGQQLTGANK | 1531.73 | 15 | non-toxin | non-allergen | stable | AMP |
| SLKDYTVADEAAELAK | 1723.9 | 16 | non-toxin | non-allergen | stable | NAMP |
| VTQGSINFAKSVAENYK | 1856.07 | 17 | non-toxin | non-allergen | stable | AMP |
| MIVTDIAGTTRDAIDSK | 1807.05 | 17 | non-toxin | non-allergen | stable | NAMP |
| AGTSFTIGSFNGDGWNSIK | 1959.1 | 19 | non-toxin | non-allergen | stable | AMP |
| KMDLAELK | 947.16 | 8 | non-toxin | non-allergen | stable | AMP |
| AEGYEVLAAAK | 1121.26 | 11 | non-toxin | non-allergen | stable | NAMP |
| DLLIDAQDVQK | 1257.41 | 11 | non-toxin | non-allergen | stable | AMP |
| LSLNVDKENPR | 1284.43 | 11 | non-toxin | non-allergen | stable | NAMP |
| MTNGLGTLQTK | 1163.35 | 11 | non-toxin | non-allergen | stable | NAMP |
| SASSTFNANAAR | 1196.24 | 12 | non-toxin | non-allergen | stable | NAMP |
| VSDNQGLMNVAR | 1303.46 | 12 | non-toxin | non-allergen | stable | NAMP |
| RMADNVYAHLAN | 1374.54 | 12 | non-toxin | non-allergen | stable | NAMP |
| QVDIPAMLVATR | 1313.58 | 12 | non-toxin | non-allergen | stable | NAMP |
| TNEDPYTIDVES | 1382.4 | 12 | non-toxin | non-allergen | stable | AMP |
| MLKAGLRVEVDER | 1515.79 | 13 | non-toxin | non-allergen | stable | NAMP |
| TYSALGEPGRMQEK | 1566.75 | 14 | non-toxin | non-allergen | stable | NAMP |
| DGSISIVDHGTEIAR | 1569.69 | 15 | non-toxin | non-allergen | stable | NAMP |
| ENGLGNYPVALTDDAR | 1704.81 | 16 | non-toxin | non-allergen | stable | NAMP |
| NVDDASNDVTFSALRK | 1751.87 | 16 | non-toxin | non-allergen | stable | NAMP |
| KENFVADEK | 1079.17 | 9 | non-toxin | non-allergen | stable | NAMP |
| KMDIGKAGDK | 1062.25 | 10 | non-toxin | non-allergen | stable | NAMP |
| LTQGIAADGR | 1001.11 | 10 | non-toxin | non-allergen | stable | NAMP |

**S1.3. Table: Predicted peptide structure values obtained from I-TASSER and PEP-FOLD3**

| **Sl.no** | **Peptide Name** | **C-score (-5 to 2) (I tasser)** | **sOPEP (pep fold3)** |
| --- | --- | --- | --- |
| 1 | VTDLDLTAEVVK | -0.57 | - |
| 2 | FENHAVEVDELSR | -0.76 | - |
| 3 | LGADATVPFDMTTK | -1.55 | - |
| 4 | KSSLVTGQQLTGANK | -1.69 | - |
| 5 | VTQGSINFAKSVAENYK | -0.63 | - |
| 6 | AGTSFTIGSFNGDGWNSIK | -1.65 | - |
| 7 | DLLIDAQDVQK | -0.08 | - |
| 8 | TNEDPYTIDVES | -0.79 | - |
| 9 | LSNRAAFFR | - | -10.7715 |
| 10 | KMDLAELK | - | -12.0533 |

**S1.4. Table: Predicted peptide structures generated by I-TASSER and PEP-FOLD3**

| **Sl.no** | **Peptide Name** |
| --- | --- |
| 1 | VTDLDLTAEVVK |
| 2 | FENHAVEVDELSR |
| 3 | LGADATVPFDMTTK |
| 4 | KSSLVTGQQLTGANK |
| 5 | VTQGSINFAKSVAENYK |
| 6 | AGTSFTIGSFNGDGWNSIK |
| 7 | DLLIDAQDVQK |
| 8 | TNEDPYTIDVES |
| 9 | LSNRAAFFR |
| 10 | KMDLAELK |

**S1.5. Table: Interaction results of MAPK3 with peptide LSNRAAFFR (A = protein; B = peptide)**

| **Non-bonded Interaction residues** | **Distance in Å** | **Type of Interaction** |
| --- | --- | --- |
| B:ARG4:HH11 - A:GLU50:OE2 | 2.21 | Hydrogen Bond;Electrostatic (Salt Bridge) |
| B:ARG9:NH1 - A:GLU203:OE2 | 4.90 | Electrostatic |
| A:ALA52:HN - B:ARG4:O | 2.34 | Hydrogen Bond |
| A:ARG84:HE - B:PHE7:O | 2.88 | Hydrogen Bond |
| A:ARG84:HH21 - B:PHE7:O | 2.35 | Hydrogen Bond |
| B:ASN3:HD22 - A:ASP128:OD2 | 2.28 | Hydrogen Bond |
| B:PHE8:HN - B:ALA6:O | 2.45 | Hydrogen Bond |
| B:ARG9:C - B:ALA6:O | 3.61 | Hydrogen Bond |
| A:ARG84:CD - B:PHE7 | 3.85 | Hydrophobic (Pi-Sigma) |
| B:ARG4 - B:LEU1 | 5.06 | Hydrophobic (Alkyl) |
| B:ALA5 - B:LEU1 | 5.19 | Hydrophobic (Alkyl) |
| B:PHE7 - A:ALA52 | 4.96 | Hydrophobic (Pi-Alkyl) |
| B:PHE7 - A:ARG84 | 4.63 | Hydrophobic (Pi-Alkyl) |
| B:PHE8 - A:ALA52 | 4.25 | Hydrophobic (Pi-Alkyl) |

**S1.6. Table: Interaction results of MAPK3 with peptide TNEDPYTIDVES (A = protein; B = peptide)**

| **Non-bonded interaction residues** | **Distance in Å** | **Type of interaction** |
| --- | --- | --- |
| A:LYS32:HZ1 - B:ASP9:OD2 | 2.32 | Hydrogen Bond; Electrostatic (Salt Bridge) |
| A:LYS32:HZ3 - B:ASP9:OD2 | 2.70 | Hydrogen Bond; Electrostatic (Salt Bridge) |
| A:LYS131:HZ2 - B:GLU11:OE2 | 2.37 | Hydrogen Bond; Electrostatic (Salt Bridge) |
| A:ARG211:HH22 - B:ASP4:OD2 | 3.02 | Hydrogen Bond; Electrostatic (Salt Bridge) |
| A:ARG208:NH1 - B:ASP4:OD2 | 5.26 | Electrostatic |
| A:ARG208:NH2 - B:GLU3:OE2 | 5.04 | Electrostatic |
| A:ARG208:NH2 - B:ASP4:OD1 | 4.07 | Electrostatic |
| A:GLU50:HN - B:ASP9:O | 2.69 | Hydrogen Bond |
| A:ALA52:HN - B:TYR6:O | 2.69 | Hydrogen Bond |
| A:LYS168:HZ1 - B:TYR6:OH | 2.80 | Hydrogen Bond |
| A:THR207:HN - B:TYR6:OH | 2.56 | Hydrogen Bond |
| A:THR207:HG1 - B:TYR6:OH | 2.02 | Hydrogen Bond |
| A:ARG208:HN - B:ASP4:OD2 | 2.39 | Hydrogen Bond |
| A:ARG208:HE - B:ASP4:OD2 | 2.24 | Hydrogen Bond |
| A:ARG211:HH12 - B:ASN2:OD1 | 2.07 | Hydrogen Bond |
| A:ARG211:HH21 - B:ASN2:OD1 | 2.30 | Hydrogen Bond |
| A:ARG211:HH21 - B:GLU3:O | 2.36 | Hydrogen Bond |
| A:TYR250:HN - B:THR1:OG1 | 2.36 | Hydrogen Bond |
| B:ASN2:HD22 - A:TYR204:O | 2.98 | Hydrogen Bond |
| B:ASN2:HD22 - B:GLU3:O | 2.69 | Hydrogen Bond |
| B:GLU3:HN - B:GLU3:OE1 | 2.22 | Hydrogen Bond |
| B:TYR6:HN - B:ASP4:O | 2.33 | Hydrogen Bond |
| B:TYR6:HH - A:ASP166:OD1 | 2.30 | Hydrogen Bond |
| B:ILE8:HN - B:TYR6:O | 2.54 | Hydrogen Bond |
| B:VAL10:HN - B:ILE8:O | 2.61 | Hydrogen Bond |
| A:GLY49:CA - B:ASP9:O | 3.71 | Hydrogen Bond |
| B:ILE8:CA - A:GLU50:O | 3.02 | Hydrogen Bond |
| A:LYS168:NZ - B:TYR6 | 4.28 | Electrostatic (Pi-Cation) |
| A:TYR130 - B:VAL10 | 5.25 | Hydrophobic (Pi-Alkyl) |
