# Supplementary material for: Identification and 3D modeling of bioactive peptides from Lactobacillus brevis RAMULAB49 protein hydrolysate with in silico ERK1 phosphorylation inhibition activity targeting diabetic nephropathy
Source: PLoS One. 2025 Sep 22;20(9):e0331192. doi: 10.1371/journal.pone.0331192 (PMC12453238; doi:10.1371/journal.pone.0331192)
Supplement: S2 File — (DOCX) [file pone.0331192.s002.docx]

**S2.1. Figure: Chromatograms of peptide profiling**

**
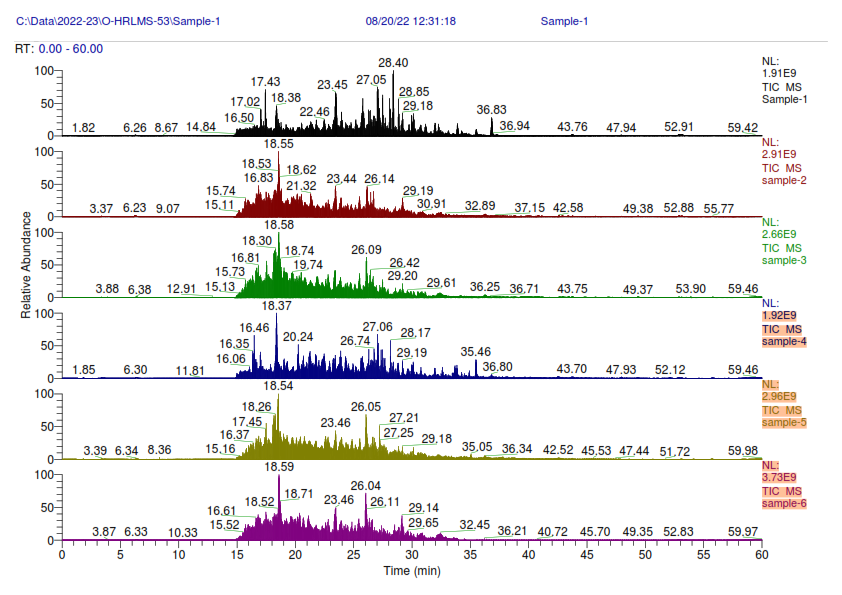
**

**S2.2. Figure: Liquid chromatography method details**

**
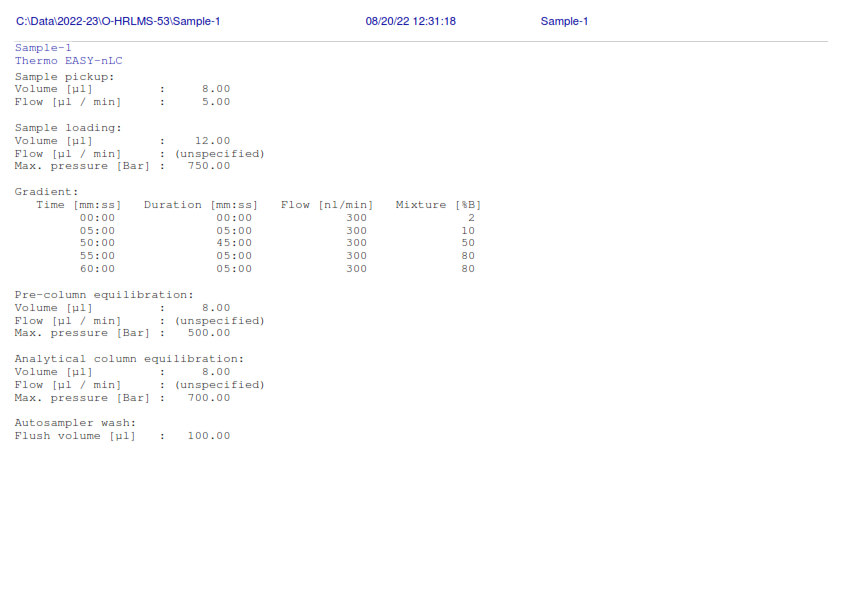
**

**S2.3. Figure: Mass spectrometry method details**

**
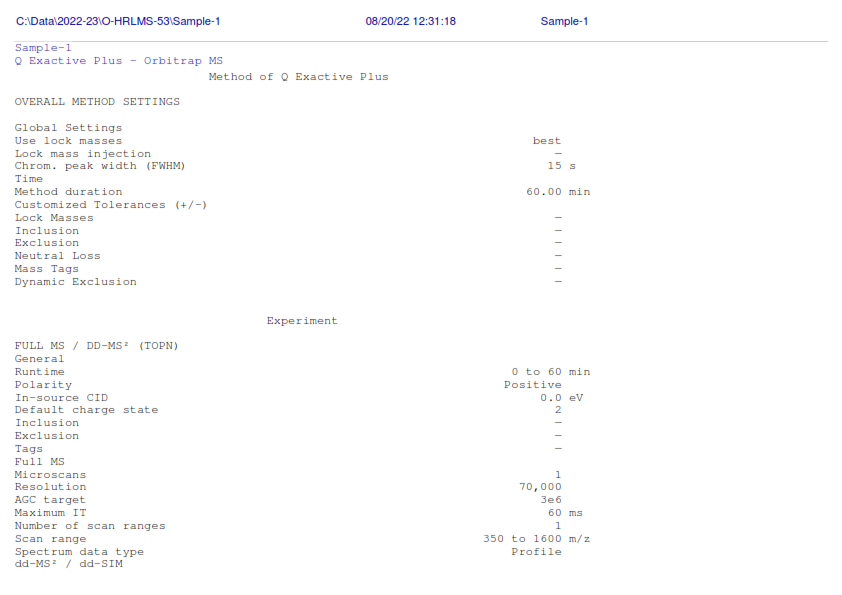
**

**
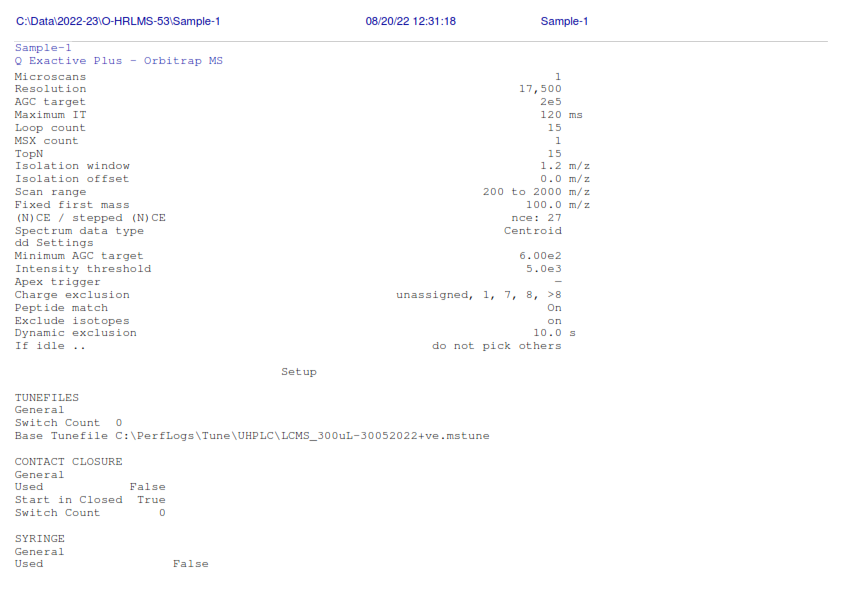
**

**
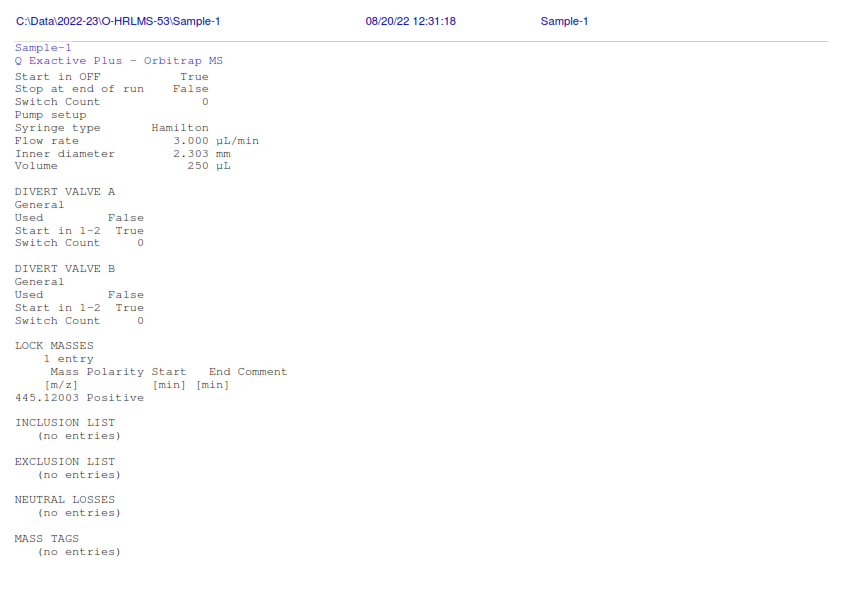
**

**S2.1. Table: Detailed peptide identification results**

| **Peptide Sequence** | **Protein** | **Sample code** |
| --- | --- | --- |
| VTDLDLTAEVVK | DEAD/DEAH box helicase family protein OS=*Lactobacillus panisapium* OX=2012495 GN=GYM71_05080 PE=4 SV=1 | Sample-6 |
| FENHAVEVDELSR | Transcription-repair-coupling factor OS=*Streptococcus thermophilus* OX=1308 GN=mfd PE=3 SV=1 | Sample - 4 |
| LGADATVPFDMTTK | Alcohol dehydrogenase OS=*Companilactobacillus alimentarius* DSM 20249 OX=1423720 GN=LA20249_07245 PE=4 SV=1 | Sample - 4 |
| KSSLVTGQQLTGANK | PTS transporter subunit EIIA OS=*Ligilactobacillus saerimneri* OX=228229 GN=GTO87_08665 PE=4 SV=1 | Sample - 4 |
| VTQGSINFAKSVAENYK | GRAM_POS_ANCHORING domain-containing protein OS=*Limosilactobacillus reuteri* OX=1598 GN=HF82_03830 PE=4 SV=1 | Sample - 6 |
| AGTSFTIGSFNGDGWNSIK | DUF5776 domain-containing protein OS=*Apilactobacillus kunkeei* OX=148814 GN=RZ73_02060 PE=4 SV=1 | Sample - 4 |
| DLLIDAQDVQK | Uncharacterized protein OS=*Secundilactobacillus folii* OX=2678357 GN=GM612_05755 PE=4 SV=1 | Sample - 5 |
| TNEDPYTIDVES | Uncharacterized protein OS=*Companilactobacillus bobalius* OX=2801451 GN=LKACC16343_01106 PE=4 SV=1 | Sample - 6 |
| LSNRAAFFR | Acetyl-CoA carboxytransferase OS=*Secundilactobacillus mixtipabuli* OX=1435342 GN=accA PE=4 SV=1 | Sample - 4 |
| KMDLAELK | Membrane protein OS=*Dellaglioa algida* OX=105612 GN=LABALGLTS371_07070 PE=4 SV=1 | Sample - 6 |

**S2.2. Table: Data for Sample 4 (pepsin)**

**S2.3. Table: Protein identification results for Sample 4 (pepsin)**

**S2.4. Table: Data for Sample 5 (pepsin and pancreatin)**

**S2.5. Table: Protein identification results for Sample 5 (pepsin and pancreatin)**

**S2.6. Table: Data for Sample 6 (pepsin, pancreatin, and trypsin)**

**S2.7. Table: Protein identification results for Sample 6 (pepsin, pancreatin, and trypsin)**
