# Supplementary material for: Identification and 3D modeling of bioactive peptides from Lactobacillus brevis RAMULAB49 protein hydrolysate with in silico ERK1 phosphorylation inhibition activity targeting diabetic nephropathy
Source: PLoS One. 2025 Sep 22;20(9):e0331192. doi: 10.1371/journal.pone.0331192 (PMC12453238; doi:10.1371/journal.pone.0331192)
Supplement: Attachment — Submitted filename: Reviewer_plos_one.docx [file pone.0331192.s004.docx]

**Reviewer #1:**

1. The manuscript identifies peptides that can bind to ERK1. Although the software is used to analyze whether these peptides have phosphorylation inhibition activity to align with the central article's main theme, it is still necessary to experimentally validate the effect of these active peptides on activity after binding to ERK1. Otherwise, despite the ability to predict the potential through software, it does not prove the impact on ERK1.

Author response: We sincerely appreciate the reviewer’s insightful comment. We acknowledge that experimental validation is crucial to confirm the inhibitory effect of the identified peptides on ERK1 activity. While this study primarily focuses on computational screening and prediction, future work is planned to include in vitro kinase inhibition assays and phosphorylation studies to validate the bioactivity of the peptides against ERK1. In the revised manuscript, we have explicitly acknowledged this limitation in the conclusion to emphasize the need for further experimental validation to confirm the functional inhibition of ERK1 activity by the identified peptides.

2. The length of MD, 100 ns, is much less than the current standards (close to 1 microsecond), so the authors might discuss their choice: probably, as the peptides are very short, their interactions with the target are adequately sampled even at 100 ns simulation time.

Author response: We sincerely thank the reviewer for the valuable suggestion. We agree that longer molecular dynamics (MD) simulations, close to the microsecond scale, are increasingly considered standard for studying larger and more complex systems. In this study, however, the 100 ns simulation length was selected considering the small size of the peptides (8–12 amino acids) and the localized nature of the binding interactions with the target protein ERK1. For short peptides, several previous reports have demonstrated that equilibrium behavior, conformational flexibility, and stable binding interactions are often adequately sampled within 50–100 ns simulation timeframes. In our case, key parameters such as RMSD, Rg, SASA, and hydrogen bonding analyses indicated convergence and stability well within the 100 ns window, supporting the sufficiency of the simulation time for this system. We have also added a clarification on this point in the revised manuscript to provide better context for readers.

Nevertheless, we acknowledge that longer simulations could provide additional insights into slow conformational transitions and rare events. Therefore, in future work, we plan to extend our molecular dynamics simulations to the microsecond scale to further validate and strengthen our findings.

3. The introduction presents the relevance of diabetic nephropathy (DN) and the need for new therapies. The authors should broaden the discussion on the limitations of current treatments and how bioactive peptides offer an advantage over existing approaches.

Author response: We thank the reviewer for this valuable suggestion. In response, we have revised the Introduction (with proper references and citations) to expand on the limitations of current pharmacological therapies for diabetic nephropathy, including their insufficient efficacy in halting disease progression and associated side effects. Additionally, we have included a detailed discussion (with proper references and citations) on the therapeutic potential of bioactive peptides, highlighting their natural origin, target specificity, low toxicity, and ability to modulate multiple pathways such as ERK1 involved in DN. These revisions aim to provide a clearer justification for the focus and relevance of our study.

4. The preparation of Lactobacillus brevis RAMULAB49 protein hydrolysates is adequately described, but have you considered optimizing hydrolysis conditions (time, enzyme concentration) to maximize the release of bioactive peptides?

Author response: As per the reviewer suggestion, we have revised the methodology to incorporate hydrolysis conditions for enhanced optimization of bioactive peptide release. The authors have performed the enzymatic hydrolysis based on their published protocol (<http://dx.doi.org/10.1016/j.xpro.2025.103657>) and the same has been cited in the manuscript.

5. Peptide identification using nLC-ESI and MS/MS is a standard. How did you manage the complexity of MS/MS data to ensure accurate peptide identification, avoiding false positives or negatives? Have the authors quantified the peptides identified? Knowing their relative abundance could help prioritize the most promising candidates.

Author response: We thank the reviewer for the insightful comment. To ensure accurate peptide identification and minimize false positives, we applied stringent parameters during MS/MS analysis using BioPharma Finder Software 2.0, applying a mass error tolerance of <5.0 ppm and a confidence level greater than 95% for peptide matches. The rest of the details have been given in the manuscript. Although we did not perform peptide quantification in this study, we acknowledge the importance of relative abundance in prioritizing peptides. Future work will incorporate label-free quantification methods to support candidate selection and functional validation.

6. Molecular docking and molecular dynamics simulation are appropriate methodologies. Did you flexibly dock both the peptide and the protein to account for the conformational changes induced by the binding? What metrics did you use to evaluate the quality and reliability of the docking models?

Author response: We thank the reviewer for this valuable comment. As described in the revised Methods section (2.7 Molecular Docking), we employed HADDOCK v2.4 for docking, which incorporates semi-flexible docking by allowing conformational changes in both the peptide and the protein at the interface during the refinement stage. This accounts for induced fit and improves the biological relevance of the docking results. To evaluate docking quality and reliability, we used HADDOCK scoring metrics including van der Waals energy, electrostatic energy, de-solvation energy, buried surface area, and Z-score. Additionally, molecular dynamics simulations were performed on the top-ranked peptide-ERK1 complexes to further assess the structural stability and binding behaviour over time.

7. Identifying ERK1 as a key target in constructing the PPI network raises questions about its role in the broader context of DN pathogenesis. What experimental evidence supports its potential as a therapeutic target?

Author response: We thank the reviewer for the insightful comment. ERK1 was prioritized based on its central role in the PPI network and its well-documented involvement in DN pathogenesis. Several studies have demonstrated that ERK1/2 signalling is activated in renal tissues during hyperglycaemic conditions, contributing to mesangial cell proliferation, inflammatory cytokine production, and renal fibrosis. These are key pathological features of diabetic nephropathy. Hence, targeting ERK1 is a promising strategy, and our in silico findings aim to support this therapeutic rationale. In the revised manuscript, we have now included relevant citations to support ERK1’s biological role and potential as a drug target in DN.

8. Molecular docking results suggest stable peptide binding to ERK1. What are the key interactions between peptides and ERK1 contribute to binding affinity? Have you compared the docking results with those of known ERK1 inhibitors?

Author response: We thank the reviewer for their valuable suggestion. In the revised manuscript, we have clarified the key interactions contributing to peptide-ERK1 binding affinity, including hydrogen bonding and electrostatic interactions with residues such as GLU50, ARG211, ARG84, and TYR130. We have also highlighted that the peptides bind at the same adenine-mimicking binding pocket as the co-crystallized inhibitor SCH772984 (PDB ID: 4QTB), suggesting a similar inhibitory mechanism. We have now included a discussion on how this overlapping binding site may contribute to competitive inhibition by mimicking ATP binding

9. The conclusions are too optimistic since it is only an in silico study. How do you intend to validate the results obtained in silico experimentally? What are the following steps to translate these findings into a potential therapy for DN?

Author response: We sincerely appreciate the reviewer’s insightful comment. We acknowledge that experimental validation is crucial to confirm the inhibitory effect of the identified peptides on ERK1 activity. While this study primarily focuses on computational screening and prediction, future work is planned to include in vitro kinase inhibition assays and phosphorylation studies to validate the bioactivity of the peptides against ERK1. In the revised manuscript, we have explicitly acknowledged this limitation in the conclusion to emphasize the need for further experimental validation to confirm the functional inhibition of ERK1 activity by the identified peptides

**Reviewer #2:**

1. The manuscript should have line numbers and page numbers, otherwise it is difficult to point out the details of the manuscript.

Author response: Thank you for the suggestion. Line and page numbers have now been added to the revised manuscript to facilitate easier review.

2. Abstract: The results in Abstract are primarily subjective descriptions, lacking valuable data support. After reading it, it won't leave much of an impression on the reader. Therefore, it is recommended that the authors provide more data to increase the readability of the results.

Author response: Thank you for your constructive feedback. In response, we have revised the abstract to include more specific quantitative data from our study, including peptide binding energy, molecular dynamics simulation duration, and network pharmacology outcomes. These additions aim to enhance the objectivity and clarity of our findings, making the abstract more impactful and informative for the reader.

3. Abstract: Please provide the full name of KEGG. When an abbreviation appears in the manuscript, write its full name first, and the abbreviation is written after the full name in parentheses. Subsequently, use the abbreviation consistently and do not write out the full term again.

Author response: As advised, the full name Kyoto Encyclopedia of Genes and Genomes (KEGG) has been provided at its first mention in the abstract. The abbreviation is used consistently throughout the manuscript thereafter.

4. Keywords: Should be “lactic acid bacteria Lactobacillus brevis” rather “lactic acid bacteria”.

Author response: The keywords section has been revised accordingly to include “lactic acid bacteria Lactobacillus brevis”.

5. Keywords: Should be “KEGG pathway” rather “KEGG pathway enrichment”.

Author response: The keyword has been corrected to “KEGG pathway” as suggested.

6. 1. Introduction, first paragraph: “[1][2][3]” and “[4],[5]”. Please read the submission requirements of PLOS ONE carefully and standardize the citation format of the references in the manuscript.

Author response: The citation format has been revised to comply with PLOS ONE guidelines throughout the manuscript.

7. 1. Introduction, third paragraph: These drugs including ACE inhibitors, ARBs, and statins …Please provide the full name of ACE and ARBs. In addition, there are similar errors in other parts of the manuscript, and authors are advised to check the whole manuscript carefully and correct these minor errors.

Author response: The full forms of ACE (angiotensin-converting enzyme) and ARBs (angiotensin II receptor blockers) have been provided at their first mention. The entire manuscript has been thoroughly reviewed and revised to ensure consistency in abbreviations and terminology.

8. 1. Introduction: In the introduction, the authors lack a in-depth review of the bioactive peptides from different resources. At present, there are some studies on the bioactive peptides from different resources, such as antioxidant peptides from Miiuy croaker swim bladders, antioxidant Peptides from Hizikia fusiformis, antioxidant peptides from Skipjack tuna (Katsuwonus pelamis) skins, antioxidant collagen peptides of Siberian sturgeon (Acipenser baerii) cartilages, antioxidant peptides from gelatin hydrolysate of Skipjack Tuna (Katsuwonus pelamis) bone, antioxidant peptides from protein hydrolysate of skate (Raja porosa) cartilage, bioactive peptides from Skipjack tuna cardiac arterial bulbs, antioxidant peptides from protein hydrolysate of bluefin leatherjacket (Navodon septentrionalis) skin, etc. It is suggested that the authors systematically review of these antioxidant peptides, so as to further explain the innovation, importance and significance of this study.

Author response: We sincerely thank the reviewer for this insightful suggestion. While our study specifically focuses on bioactive peptides derived from Levilactobacillus brevis RAMULAB49, we agree that a brief overview of antioxidant peptides from other natural resources would enhance the context and significance of our work. Accordingly, we have revised the introduction to include a concise summary of relevant studies on antioxidant and bioactive peptides from various sources. This inclusion helps to highlight the unique positioning of lactic acid bacteria-derived peptides and supports the novelty of our study.

(9) 2. Materials and methods: Should be “2.1 Materials and reagents” rather “2.1 Materials”.

Author response: Thank you for the observation. The subsection title has been revised to “2.1 Materials and reagents” in accordance with the suggestion.

(10) 2.1 Materials: It is recommended that the authors provide relevant parameters of pepsin, trypsin, and pancreatin, such as enzyme activity. In addition, “in vitro” should be italicized. In addition, there are similar errors in other parts of the manuscript, and authors are advised to check the whole manuscript carefully and correct these minor errors.

Author response: As per the reviewer suggestion, the authors have provided the relevant parameters of pepsin, trypsin, and pancreatin, such as enzyme activity to improve the quality of the manuscript and to be precise. *In vitro* has been italicized in the entire manuscript. All the minor errors have been justified by carefully proofreading the entire manuscript.

(11) 3.1 In vitro gastrointestinal digestion and Identification of peptides by nano-LC-MS/MS: the concentrations of the protein hydrolysates were 0.144 mg/mL, 0.875 mg/mL, and 0.747 mg/mL, respectively. It is suggested to use the conventional format "mean ± sd%" for data presentation. Review the entire text and make corrections accordingly.

Author response: As per the reviewer suggestion, the authors have provided the conventional format for data presentation in 3.1 results section. Also the entire manuscript has been read and minor errors have been rectified to improve the quality of the manuscript.

12. That brings me to the issue that the manuscript would benefit from a separate Results and Discussion section. A separate results section will also allow authors to structure their finding and resume the information. This will improve readability. A separate Discussion also allows authors to extend their interpretation. Experimental work from other groups or strategies can be discussed.

Author response: We sincerely thank the reviewer for the insightful suggestion regarding the separation of Results and Discussion sections. While we acknowledge that this restructuring can enhance clarity, we have opted to maintain a combined Results and Discussion section in the current version to preserve the continuity and direct interpretation of the in silico findings. As the study is focused on a specific peptide-based computational pipeline, we believe that the integrated format allows a coherent presentation of results alongside immediate biological relevance and interpretation. Nevertheless, we will consider this suggestion for future studies involving more extensive experimental.

**Reviewer #3:**

It would be appreciated if the authors could add more information on how the mass spectrometry analysis was performed, i.e the proteome that was downloaded for the search, the enzymes used, the modifications included, the FDR filtering of their data. I couldn’t find whether the data was downloaded onto PRIDE.

I recommend publishing with minor revisions.

Author response: We sincerely thank the reviewer for their thoughtful suggestions to improve the clarity of our mass spectrometry methodology. We have carefully revised the manuscript to include the requested details regarding the mass spectrometry analysis, database search parameters, and data availability. Below, we provide a detailed response addressing each point raised:

We have specified that the protein database used for peptide identification was the UniProt database corresponding to *Lactobacillus* spp. This has been clearly mentioned in the Methods section.

The enzymatic specificity parameters were set for pepsin and pancreatin, reflecting the digestion conditions used during sample preparation. This information has been added to clarify the digestion specificity employed during the database search process.

In our workflow, no fixed or variable modifications were set during the database search, as the objective was to map naturally occurring peptides resulting from enzymatic hydrolysis without chemical derivatization. We have now included a statement regarding modifications in the revised manuscript.

The raw data were validated using Thermo Proteome Discoverer 2.2. A fixed threshold strategy was applied, accepting only >95% confidence and q-value less than 0.01 to ensure high-confidence peptide identification. Intact mass analysis and peptide mapping were performed using BioPharma Finder Software 2.0, applying a mass error tolerance of <5.0 ppm and a confidence level greater than 95% for peptide matches.

We acknowledge the reviewer’s query regarding data deposition. At this stage, the raw mass spectrometry data have not been uploaded to public repositories such as PRIDE. This information has been mentioned in the revised manuscript. All the alterations have been made in the sections 2.3 and 2.4.
