# Supplementary material for: Identification and 3D modeling of bioactive peptides from Lactobacillus brevis RAMULAB49 protein hydrolysate with in silico ERK1 phosphorylation inhibition activity targeting diabetic nephropathy
Source: PLoS One. 2025 Sep 22;20(9):e0331192. doi: 10.1371/journal.pone.0331192 (PMC12453238; doi:10.1371/journal.pone.0331192)
Supplement: Attachment — Submitted filename: Response_new.docx [file pone.0331192.s005.docx]

**Reviewer 1 Comments**

**1) Optimisation of hydrolysis conditions:** The authors have cited their previously published protocol and indicated that they have revised the methods to align with it. However, a concise justification within the text explaining the reasons for not further optimising the conditions in this specific work (e.g., constraints, prior validation) would enhance clarity.

**Authors Response**: Authors thank the reviewer for the insightful comments. The hydrolysis conditions used in this study were adopted from our previously published work (<https://doi.org/10.3389/fmicb.2023.1190105>) (Reference No. 21), which demonstrated high efficiency in releasing bioactive compounds with therapeutic potential. Given the robustness and reproducibility of the established method, no additional optimisation was undertaken in this work. Moreover, maintaining the same conditions allowed us to ensure consistency with our prior findings, enabling a reliable comparison of peptide activity across studies. The decision was also influenced by time and resource constraints, and the focus of this study was on target-specific peptide screening and validation, rather than re-optimisation of hydrolysis parameters. According to the reviewer’s suggestion, the justification has been added in the methodology section (highlighted in yellow).

**2) Experimental validation of peptide effects on ERK1 activity:** The authors have appropriately acknowledged the absence of experimental validation and explicitly state this limitation in the manuscript. While the inclusion of planned in vitro kinase assays is a reasonable future direction, I recommend emphasising in the ‘Discussion’ section that computational predictions are merely suggestive and should not be misconstrued as definitive evidence of efficacy.

**Authors Response**: We thank the reviewer for the insightful comments. We fully agree that computational predictions are suggestive in nature and should not be interpreted as definitive evidence of efficacy. Accordingly, we have revised the *Discussion* section (highlighted in yellow) to more explicitly emphasize this limitation. We have also modified some content in *Introduction* to match the same from *Discussion*. We have clarified that the current study is intended to serve as a preliminary screening framework to identify a DN-specific target (ERK1) and to prioritize a lead peptide for future experimental validation. We have also reiterated that *in vitro* and *in vivo* studies are necessary to confirm the biological relevance and therapeutic potential of the findings.

**Reviewer 2 Comments**

1) The authors have answered my questions well and made the necessary changes to the manuscript (ID: PONE-D-25-05892R1). It looks ready for publication as far as I can tell. Then, I think that the manuscript can be accepted for publication in PLOS ONE.

**Authors Response**: We sincerely thank the reviewer for their positive feedback, thoughtful evaluation, and recommendation for acceptance. We are grateful for the time and effort invested in reviewing our manuscript, and we truly appreciate the constructive comments that helped us improve the quality and clarity of our work. We are pleased to hear that the revised version meets the expectations and is considered for publication.

**Reviewer 3 Comments**

1) Thank you for addressing all the comments. I would still have liked to be able to see the actual raw data on PRIDE to review the mass spec data.

**Authors Response**: The authors appreciate the reviewer’s concern regarding the transparency and accessibility of raw mass spectrometry data.

This study involved a targeted peptidomics workflow designed solely to identify bioactive peptides from *Lactobacillus brevis* RAMULAB49 protein hydrolysates for downstream in silico modeling and docking studies. The LC–MS–ESI analyses were performed at SAIF, IIT Mumbai, using a Thermo Fisher Scientific Q-Exactive Plus Biopharma High-Resolution Orbitrap system. At the time of acquisition, the RAW vendor files were made available via a temporary secure link, which was accessed and reviewed for quality verification from both the ends (Our university and SAIF, IIT Mumbai).

However, in routine targeted peptidomics studies such as this, the validated processed outputs generated by the Thermo Proteome Discoverer and BioPharma Finder pipelines were provided by the SAIF IIT, Mumbai were considered the final authenticated deliverables, as they had already incorporated the following:

•	High-confidence peptide assignments with stringent quality filters (mass accuracy < 5 ppm, >95% confidence, q < 0.01)

•	Removal of low-quality or ambiguous spectra

•	Accurate protein/peptide mapping with enzymatic specificity

Therefore, only the processed data provided by SAIF, IIT Mumbai has been used in this study, as it sufficiently captures the experimental outcomes necessary for peptide identification and all downstream bioinformatic analyses.

Recently, in response to the reviewer’s request, we contacted SAIF to provide the original RAW files for PRIDE deposition. However, the facility informed us that their standard retention policy archives RAW spectra for only one year, and the files from this 2023 analysis are no longer available.

To ensure full transparency and reproducibility of our work, we are providing all available processed data as supplementary materials, compiled in a single ZIP archive:

LC and MS acquisition method details, along with representative chromatograms to verify data quality and experimental validity, Original peptide and protein identification tables for each enzymatic treatment condition (pepsin, pepsin + pancreatin, pepsin + pancreatin + trypsin).

Although the dataset cannot now be deposited in PRIDE due to the unavailability of the RAW files, these curated outputs are scientifically sufficient to reproduce the peptide identifications and support the conclusions of this manuscript.

We sincerely thank the reviewer for highlighting this important point. Moving forward, we will ensure long-term archiving of vendor RAW files and PRIDE deposition in all future mass spectrometry-based studies to enhance data accessibility for the wider scientific community.
